# A Comprehensive Analysis of Northern versus Liquid Hybridization Assays for mRNAs, Small RNAs, and miRNAs Using a Non-Radiolabeled Approach

**DOI:** 10.3390/cimb43020036

**Published:** 2021-06-22

**Authors:** Waqar Ahmad, Bushra Gull, Jasmin Baby, Farah Mustafa

**Affiliations:** Department of Biochemistry, College of Medicine and Health Sciences, United Arab Emirates (UAE) University, Al Ain 20000, United Arab Emirates; waqar.ahmad@uaeu.ac.ae (W.A.); 201790692@uaeu.ac.ae (B.G.); 201790693@uaeu.ac.ae (J.B.)

**Keywords:** northern blotting, liquid hybridization assay, exonuclease 1, non-radioactive, biotinylation, mRNA, small RNA, miRNA, Typhoon & Sapphire biomolecular imaging

## Abstract

Northern blotting (NB), a gold standard for RNA detection, has lost its charm due to its hands-on nature, need for good quality RNA, and radioactivity. With the emergence of the field of microRNAs (miRNAs), the necessity for sensitive and quantitative NBs has again emerged. Here, we developed highly sensitive yet non-radiolabeled, fast, economical NB, and liquid hybridization (LH) assays without radioactivity or specialized reagents like locked nucleic acid (LNA)- or digoxigenin-labeled probes for mRNAs/small RNAs, especially miRNAs using biotinylated probes. An improvised means of hybridizing oligo probes along with efficient transfer, cross-linking, and signal enhancement techniques was employed. Important caveats of each assay were elaborated upon, especially issues related to probe biotinylation, use of exonuclease, and bioimagers not reported earlier. We demonstrate that, while the NBs were sensitive for mRNAs and small RNAs, our LH protocol could efficiently detect these and miRNAs using less than 10–100 times the total amount of RNA, a sensitivity comparable to radiolabeled probes. Compared to NBs, LH was a faster, more sensitive, and specific approach for mRNA/small RNA/miRNA detection. A comparison of present work with six seminal studies is presented along with detailed protocols for easy reproducibility. Overall, our study provides effective platforms to study large and small RNAs in a sensitive, efficient, and cost-effective manner.

## 1. Introduction

Recent advances in RNA biology and techniques to study gene expression at the transcription level have witnessed unparalleled progress, with our understanding of different forms of RNAs being constantly challenged. The advent of comprehensive sequencing techniques has revealed that more than 80% of the total genome is extensively transcribed, with only a mere 1–2% of the transcripts coding for functional proteins [1]. This highlights the regulatory nature of the remaining proportion of RNA molecules transcribed from “junk DNA” or regions that do not code for any protein. These RNA species are now found to be central regulators of almost all cellular processes, including gene expression, cell growth, proliferation, and differentiation. The first non-protein-coding small RNA species were identified in *C. elegans* and were observed to be pivotal for their development [2]. These regulatory molecules termed microRNAs (miRNAs), have since become the most extensively studied and well-characterized type of small non-coding RNAs, implicated in every aspect of gene expression [3]. With further characterization and understanding of small RNAs, miRNAs were observed to be only the first of many types of non-coding regulatory RNAs, including, long non-coding RNAs [4] and piRNAs [4,5]. miRNAs are synthesized through multiple enzymatic cleavage reaction by RNase-III-like enzymes, such as Drosha and Dicer [6,7,8,9], important for crucial cellular pathways, including cell development and differentiation [10,11], cell cycle regulation [12], and cell proliferation [13]. Consequently, dysregulation of their expression leads to cell transformation and promotion of carcinogenesis [10,14,15,16,17]. Furthermore, these regulatory RNAs have been observed to function as both a friend and a foe of intracellular pathogens such as viruses [18,19,20,21,22].

Molecular characterization of a protein-coding gene generally involves comprehensive analysis of its relative RNA expression. Northern blotting was considered the gold standard among well-established analytical techniques traditionally used for the detection of a particular RNA moiety in a pool of total RNA using size fractionation [23,24]. It is a relatively simple and semi-quantitative technique that can be used to evaluate gene expression at different times, between different tissues, or across species, but at the same time, has low sensitivity without the use of radioactivity and lacks high throughput, making it inappropriate for large-scale gene expression analysis. Therefore, other techniques such as sequencing, microarrays, and PCR-based approaches like real-time PCR have stepped in to resolve these limitations [25,26]. Furthermore, these newer techniques have greatly facilitated the discovery and quantitation of the small non-coding group of RNAs. However, these approaches do not differentiate amongst the primary, precursor and mature forms of miRNAs which is critical to study their biogenesis [27]. Thus, scientists have reverted to the classical and improved versions of the northern blotting technique for the identification, biogenesis, and validation of small regulatory RNA expression. Northern blotting is a more promising approach for small RNA study since it displays the size distribution of miRNA precursors and mature forms, ranging from ~1000 nucleotides (nt) for pri-miRNAs, 120–60 nt for pre-miRNAs, and 20–25 nt for mature miRNAs [1,28,29].

**Figure 1:**
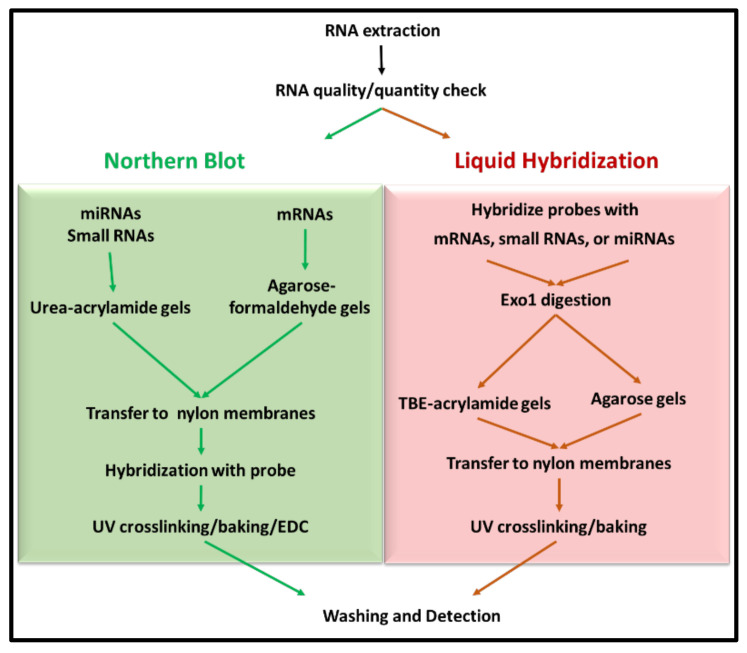
Schematic diagram showing the difference between northern blotting with the liquid hybridization methods. During northern blotting, extracted RNA is subjected to size fractionation using denaturing agarose or acrylamide gel. After fractionation, RNA is transferred to the positively charged or neutral membranes and crosslinked by UV or EDC (1-ethyl-3-(3-dimethyl aminopropyl) carbodiimide), or baked, followed by hybridization with labeled probe(s) and detection. Contrary to this method, in the liquid hybridization technique, the RNA is initially hybridized with the labeled probe and then subjected to the non-denaturing agarose or acrylamide gel and subsequently transferred to the membrane, followed by its crosslinking/baking, and detection.

Northern blotting for small RNAs involves fractionation of RNA on denaturing acrylamide gels, which is subsequently transferred onto appropriate membranes by electrophoretic transfer. Subsequently, RNA is detected by hybridization with labeled nucleic acid probes (Figure 1) [23]. The technique not only helps in determining the size of the transcript, but also identifies alternatively spliced variants of the same. However, as mentioned earlier, sensitivity has been one of the major caveats of the classical technique when non-radiolabeled probes are used. The integrity of the RNA sample used also weighs heavily on the quality of data obtained. Multiple probe analysis is another limitation associated with the technique, which requires the blot to be stripped before hybridization with a second probe. Furthermore, the entire process is tedious and requires several days before the data are obtained.

Some of the shortcomings of the classical northern blotting technique have been resolved by modifications in the probe design, labeling, and detection aspects of the technique. For example, non-radioactive probes (such as those labeled with digoxigenin (DIG), biotin, or fluorescein) have been introduced as an alternative to radiolabeled probes used conventionally [30,31,32]. These non-radioactive probes showed promising results with increased sensitivity and reduced exposure time when compared to radioactive probes [30,31]. Additionally, they save time and effort of prior security approvals, offer protection from health hazards, and eliminate radioactive disposal problems [30].

Using northern blots for small RNA molecules has further created challenges as well as opportunities for its improvement. One such improvement was introduced by Pall et al. [33], who modified the crosslinking process for small RNAs due to their weaker ability to bind to the membranes after transfer compared to mRNAs. This was achieved by using a water-soluble carbodiimide, 1-ethyl-3-(3-dimethyl aminopropyl) carbodiimide (EDC), to mediate chemical crosslinking instead of the conventional UV crosslinking process. Although the precise mechanism of crosslinking is not known, UV crosslinking is supposed to act upon the uridine base of RNA molecules, leading to the formation of reactive species. These reactive species form covalent bonds with the free amine groups of nitrocellulose and/or nylon membranes [31,33]. The involvement of nucleotide bases in the crosslinking reaction might affect subsequent hybridization of the probe by potentially hijacking the probe binding sites via modifying functional groups. This further affects complementarity and eventually results in reduced probe binding efficiency.

Other modifications have also been introduced for small RNA northerns. For example, Valoczi et al. have tested modified locked nucleic acid (LNA) probes to improve sensitivity and specificity towards mature miRNA detection by 10-folds. However, the usage of these modified probes is limited by their cost and commercial availability [34]. Kim et al., on the other hand, showcased a hybrid approach, employing the LED protocol consisting of (DIG)-labeled oligonucleotide probes with modified locked nucleic acids coupled with EDC for crosslinking the RNA to the membrane. This protocol has been shown to allow detection of very low amounts of RNA (up to 0.05 fmol) in a short exposure-time and has been shown to be a cost-effective technique compared to isotope labeling that can detect RNA at 0.4 fmol range [35]. Besides these alterations within the northern blotting protocol for small RNAs, Wang et al. have described a remarkable new approach to northerns using liquid hybridization where the small miRNAs were hybridized with non-radioactive fluorescent probes before acrylamide gel electrophoresis. This step was followed by visualization using chemiluminescent detection (Figure 1). They state that results could be obtained in a fewer hours compared with the northern technique, which takes days to complete. Additionally, the expression of multiple miRNAs could be analyzed simultaneously [32,36].

With all the different variations introduced into the classical technique of northern blotting as well as liquid hybridization, in this study, we analyzed the different modifications and permutations introduced and used them to develop highly sensitive, much cheaper, and rapid liquid hybridization assays for not just one but three main categories of RNAs: the large class of mRNAs, the intermediate small RNAs, and the tiny miRNAs using easily available, non-radiolabeled, biotinylated probes. In parallel, we performed a systematic analysis of these liquid hybridization assays with northern blotting, to compare their relative levels of sensitivity, specificity, and utility for each category of RNAs. Throughout the manuscript, we highlight important experimental details which are critical to the success of both techniques as well as pinpoint their caveats, especially related to the use of exonucleases for excess probe digestion and detection of non-radiolabeled signals using commonly available biomolecular imagers. Finally, we compare our results with other noteworthy studies published in this area in the last few years to emphasize the enhanced sensitivity of our protocols and contributions made by our work.

Overall, our results reveal that liquid hybridization is a much more sensitive, simpler, and faster technique than classical northern blotting for all three classes of RNAs and more amenable to larger sample size. Additionally, liquid hybridization was observed to be especially suited for the sensitive detection of miRNAs in the absence of specialized reagents compared to northern blotting. Evaluation of the sensitivity of our non-radiolabeled liquid hybridization assays with those published earlier revealed their enhanced sensitivity, making them comparable to radiolabeled assays. Thus, we present highly sensitive, rapid, and cost-effective liquid hybridization assays that do not need specialized probes like LNA or radioactivity for the detection of mRNAs, small RNAs, and miRNAs.

## 2. Materials and Methods

### 2.1. RNA Extraction

Total cellular RNA was extracted from either the normal mouse mammary cell line, HC11 (RNA1 and RNA2) or 293T cells (RNA3 and RNA4) using 500 µL of TRIzol™ Reagent (Catalogue # 15596018, Thermo Fisher Scientific, Waltham, MA, USA) according to the manufacturer’s instructions [37]. The extracted RNA pellets were resuspended in TE buffer (1 mM Tris, pH 8.0/0.1 mM EDTA, pH 8.0) or DEPC water, quantified using Nanodrop spectrophotometer, and stored at −80 °C until further use.

### 2.2. Synthesis of Oligonucleotides and Probes

All primers used in this study were synthesized commercially (Macrogen Inc., Seoul, Republic of Korea) and their sequence is provided in Table 1. Biotin labeling of the probes was accomplished either commercially by directly labeling the primers at the 5′ end (Macrogen Inc.) or in-house by using the Biotin DecaLabel DNA labeling kit (Catalogue # K0652; Thermo Fisher Scientific) according to the manufacturer’s protocol. The biotinylated primers were diluted to 50 ng/µL (10 pmol) and used as miRNA probes. Probes for mRNA were prepared via PCR-amplification from cDNAs using pre-biotinylated primers. Briefly, 6 µg total RNA was DNase-treated using Turbo DNase (Thermo Fisher Scientific) and reverse transcribed using MMLV reverse transcriptase (Promega, Madison, WI, USA), as per manufacturer’s instructions. DNA fragments for use as probes were synthesized by PCR amplification using pre-biotinylated primers at the 5′ end as follows: denaturation 94 °C, 45 s; annealing 59 °C 45 s and extension at 72 °C, 45 s, 35 cycles. The PCR amplified products were size fractionated on 2% agarose gels, followed by their gel purification (Qiagen Gel Extraction Kit (Catalogue # 28704), Hilden, Germany). The purified probes were quantified using the Nanodrop spectrophotometer and the biotinylation of the probes and primers was verified using dot blot assays prior to use.

### 2.3. Liquid Hybridization

#### 2.3.1. Sample Preparation for Liquid Hybridization: mRNA, Small RNA, and miRNA

The protocol of Wang et al. was used for liquid hybridization of mRNA, small RNA, and miRNA with a few critical modifications [32]. Briefly, purified RNAs (RNA1 and RNA2) were serially diluted two-folds starting from either 2.5 or 1.0 µg four times before use. One µL (50 ng) of the PCR-synthesized biotinylated probe was mixed with 15 µL hybridization buffer (10 mM EDTA, 0.3 M NaCl, 30 mM sodium phosphate buffer at pH 8.0) in the case of mRNAs, while for the small RNAs and miRNAs, 10 pmol of biotin-labeled primers were used for each concentration of target RNA in a final volume of 25 µL. After thorough mixing, the RNA mix was denatured at 94 °C for 10 min followed by stepwise annealing for specific time periods using a conventional thermocycler as follows: 72 °C for 30 min, 60 °C for 60 min, 55 °C for 30 min, 42 °C for 60 min, 37 °C for 30 min. This graded reduction in temperature allowed efficient annealing of the probe to the target RNA irrespective of the melting temperature of the oligonucleotide probe, thus allowing use of one hybridization program for oligonucleotide probes with different melting temperatures [32,38]. After completion of the hybridization reaction, the hybridized samples were digested with 1 unit of Exonuclease I (Exo-I, Catalogue # M0293; New England BioLabs, Ipswich, MA, USA) overnight at 37 °C. This process eliminates non-hybridized, single-stranded DNA probe in the 3′ to 5′ direction. The digested mixture was either stored at −20 °C until further use or subjected to electrophoresis for northern or analyzed by slot blot.

#### 2.3.2. Gel Electrophoresis of mRNA, small RNA, and miRNA for Liquid Hybridization

Non-denaturing, 1.2% agarose-MOPS (3-N-morpholinopropanesulfonic acid) gels with EtBr (0.05 µg/mL) were used for mRNA, while 12% TBE (Tris/Borate/EDTA)-acrylamide gels were used for small RNA and miRNA analyses. After polymerization, the gels were pre-run (running buffer: 1× MOPS for agarose and 1× TBE for acrylamide gel) for 30 min at 80 V and 150 V, respectively, followed by loading of RNA or hybridized samples mixed with 5× non-denatured RNA loading buffer. The agarose gels for mRNAs and acrylamide gels for small RNAs were run for 40–90 min at 80 V and 150 V, respectively, or until the bromophenol dye reached two-thirds of the gel.

#### 2.3.3. Membrane Transfer and Crosslinking for Liquid Hybridization

After the completion of gel electrophoresis, the acrylamide gels were stained with EtBr (0.05 µg/mL) for visualization using UVP Imager and/or Typhoon using EtBr filter. This was followed by pre-soaking the gels in either 1× MOPS (agarose gels) or 1× TBE buffer (acrylamide gels). RNA in the gels was transferred onto positively charged Hybond nylon membranes (GE Healthcare, Chicago, IL, USA) pre-soaked in the same buffer used for equilibration. Electroblotting was performed at 80 mA constant current for 1 h for agarose gels and 18 mA for 1 h for acrylamide gels, respectively. After the completion of the transfer, membranes were placed on pre-soaked filter papers with either 1× MOPS or 1× TBE buffers and crosslinked using UV crosslinker at 700 µJ (Stratagene, San Diego, CA, USA). This was followed by baking at 80 °C for two hours. After crosslinking and baking, membranes were wrapped and stored at −20 °C. This was followed by signal detection and imaging which will be discussed commonly for both northern blotting and liquid hybridization techniques in Section 2.6.

### 2.4. Northern Blotting

#### 2.4.1. Gel Electrophoresis for Northern Blots: mRNAs, Small RNAs, and miRNAs 

A 1.2% denaturing agarose-formaldehyde gel containing EtBr (0.05 µg/mL) was prepared for mRNAs, whereas 15% denaturing 7 M urea-acrylamide gels were prepared for small RNAs and miRNAs. Samples were prepared after a two-fold sequential dilution of both RNA samples, but starting from 25 μg RNA for northern blots, which is ten times the amount that was used in the liquid hybridization experiments. Thus, the RNA samples were diluted from 25 µg to 12.5 µg, 6.25 µg till 3.125 µg, respectively, and mixed with 2× denaturing RNA loading dye prior to loading. The running buffer used for agarose gels was 1× MOPS containing 0.1 M formaldehyde, while 1× TBE buffer was used for the 7M urea-acrylamide gels. Similar conditions for pre-run and run were followed for northern blotting, as described earlier, for liquid hybridization.

#### 2.4.2. Membrane Transfer and Crosslinking for Northern Blots

After the completion of gel electrophoresis, the acrylamide gels were stained with EtBr (0.05 µg/mL) for visualization using UVP Imager and/or Typhoon using EtBr filter. This was followed by pre-soaking the gels in either 1× MOPS (agarose gels) or 1× TBE buffer (acrylamide gels). RNA in the gels was transferred onto positively charged Hybond nylon membranes (GE Healthcare, Chicago, IL USA) pre-soaked in the same buffer used for equilibration. Electroblotting was performed at 80 mA constant current for 1 h for agarose gels and 18 mA for 1 h for acrylamide gels, respectively. After the completion of the transfer, membranes were placed on pre-soaked filter papers with either 1× MOPS or 1× TBE buffers and first UV crosslinked at 700 µJ. This was followed by chemical crosslinking by EDC rather than baking, as described earlier [33]. Briefly, a filter paper soaked in a freshly prepared solution of EDC was placed on a plastic wrap and the UV-cross-linked membrane was placed upside down on the soaked filter paper and wrapped properly. This was followed by incubation at 60 °C for 2 h. After chemical crosslinking, the membranes were washed prior to pre-hybridization or dried and stored at −20 °C for later use.

#### 2.4.3. Hybridization of Nylon Membranes with mRNA, Small RNA or miRNA Probes

After crosslinking, the membranes were prepared for hybridization with different probes. A modified Church Buffer was used as the hybridization buffer [35,39]. The membranes were subjected to pre-hybridization in 10 mL of the hybridization buffer at 60 °C for 4 h for mRNAs and 46–48 °C for 5 h for small RNAs and miRNAs, respectively, followed by overnight hybridization as described. For mRNA detection, 1 µg of the biotinylated PCR-synthesized probe was mixed with 90 µL of 10 mM EDTA and heat-denatured at 95 °C for 10 min followed by snap cooling on ice for 5 min. The prepared probes were added directly to 10 mL of pre-hybridization buffer and mixed thoroughly. The mRNA probes were hybridized at 60 °C overnight (12–16 h) in a shaking incubator at 70 rpm. For small RNAs and miRNAs, 500 pmol of single-stranded DNA oligo probes were added to 500 µL of pre-heated hybridization buffer without any prior heat denaturation. The prepared probes were added to 10 mL of the pre-hybridization buffer and hybridized at 45–48 °C overnight in a shaking incubator at 70 rpm. The optimal hybridization temperatures varied for small RNA and miRNA probes based on the hybridization buffers used and their respective annealing temperatures. Accordingly, they should be determined empirically. All probe/primer sequences are provided in Table 1.

### 2.5. Membrane Blocking and Biotin-Streptavidin-HRP Binding

The membranes from liquid hybridization samples were washed twice with high stringency buffer containing 0.1× SSC and 0.1% SDS for 5 min to remove residual buffers or contaminants prior to blocking, while the membranes from northern blotting were first washed twice in a low stringency buffer containing 2× SSC and 0.1% SDS for 15 min and then twice for 5 min with the high stringency buffer. After washing, membranes were blocked in a 25 mL blocking buffer containing 0.1% casein and 0.1% TBST for 60 min. Blocking was followed by incubation of the membranes with diluted horseradish peroxidase (HRP)-conjugated polystreptavidin protein (Catalogue # 21140; Thermo Fisher Scientific) at room temperature in the blocking buffer. The HRP-conjugated streptavidin was used at a dilution of 1:300, 1:1000, or 1:3000 for miRNA, small RNA, and mRNA, respectively. After a 30-min incubation, the membranes were washed in the washing buffer (1× TBS/0.1% SDS) buffer for 30 min at room temperature and then either subjected to signal detection or stored at −20 °C.

### 2.6. Signal Detection Using X-ray, Typhoon or Sapphire Biomolecular Imager

The HRP signals on the hybridized membranes were detected using the ECL chemiluminescent detection kit, as per manufacturer’s directions (Catalogue # 32209; Thermo Fisher Scientific) or the SuperSignal^TM^ West Femto Maximum Sensitivity Substrate (Catalogue # 34094; Thermo Fisher Scientific). The probe signal was detected by either exposing the membranes to an X-ray film in a darkroom or by using the biomolecular imager Typhoon FLA95000 (GE Healthcare, Piscataway, NJ, USA) by using the ECL plus filter or Sapphire (Azure Biosystems, Dublin, CA, USA). Exposure times varied between 5–30 s for the X-ray films and Typhoon biomolecular imager, depending upon the probe and kit used, and 2 min for the Sapphire biomolecular imager. The femto substrate was used only for experiments where the probe amounts were low due to purification after biotinylation on acrylamide gels.

### 2.7. Stripping and Reuse of Membranes

After northern blotting, the resultant nylon membranes were stripped by washing two or more times in a pre-boiled 0.5% SDS solution for 10 min in a benchtop rocker. The membranes were then sealed in plastic pouches containing 0.5% SDS solution and incubated overnight at 60 °C in a shaking incubator. After overnight stripping, the membranes were washed once with 2× SSC solution and confirmed for lack of signal from the previous probe by exposing the membranes to an X-ray film for at least 45 min and re-stripped, if needed, or stored at −20 °C, or re-hybridized with a different probe. Membranes with no signal from the previous probe(s) were pre-hybridized with modified Church Buffer and the blotting procedure was repeated as stated above. The membranes prepared after liquid hybridization cannot be stripped. Hence, an individual hybridization process was conducted for every probe used.

### 2.8. Slot Blotting of Liquid Hybridized Probes

The sensitivity of the probes for liquid hybridized was tested using the slot blot technique. For slot blots, the Exo-I digested probes after liquid hybridization were mixed with pre-chilled 200 µL of denaturation solution (10 mM EDTA and 0.1 N NaOH). The membranes were placed into the slot blot (Bio-Rad) according to the manufacturer protocol and washed twice with chilled denaturation solution. Samples were transferred to the membranes by applying a vacuum followed by washing with denaturation solution. After washing, the membranes were removed from the slot blot and probes were crosslinked by UV crosslinker and 2-h baking at 80 °C. The crosslinked membranes were subjected to blocking, streptavidin-HRP incubation, and signal detection using the procedures described earlier.

### 2.9. Quantitative Analysis of Data

The intensity of signals produced after mRNA, small RNA, or miRNA probing was measured/quantified using GelQuant.Net 1.8.2 software. GraphPad Prism 7 was used to create the graphs.

## 3. Results

**Figure 2:**
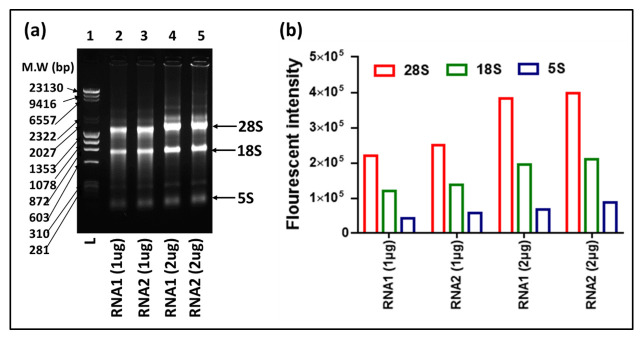
Analysis of RNA quality from the mouse mammary epithelial cell line HC11 using ethidium bromide staining. TRIzol-extracted RNA samples from cell lines were quantified using nanodrop and electrophoresed on 1% (*w*/*v*) agarose gels. (**a**) Ethidium bromide-stained gel image with 1 and 2 µg of RNA. (**b**) Quantification of the ratio of 28S, 18S, and 5S RNA bands using Gelquant.NET.

An ideal northern blot result primarily depends on the quality and integrity of the extracted RNA. Although multiple protocols have been described for RNA extraction, RNA extracted through TRIzol is still preferable for molecular biology studies, especially when it comes to the isolation of all classes of RNAs, from the larger ribosomal and mRNAs in the kilobase range, to the small RNAs in the 100 to 200 nucleotide (nt) range, to the miRNAs in the 20–25 nt range. Thus, in this study, RNA from the mouse mammary epithelial cell line HC11 was extracted through TRIzol that revealed the three distinct expected bands for 28S, 18S, and 5S RNAs (Figure 2). Our results showed a ratio for RNA1 (1 ug) of 28S:18S:5S = 1.84:1.00:0.35, and for RNA2 (1 μg) = 1.82:1.00:0.42. Doubling the amount of RNA also doubled the band intensity, keeping approximately the same ratio between 28S:18S:5S (RNA1 (2 ug)) = 1.96:1.00:0.34, and for RNA2 (2 μg) = 1.89:1.00:0.42). These data show that our analyses started out with good quality RNA since a ratio of 2:1 for 28S:18S is considered as good quality of RNA purified via cesium chloride centrifugation necessary for northern blot analysis [40,41]. It also showed that the ratio of 18S:5S for good quality RNA should be ~1:0.3–0.4 (Figure 2), something to consider when one is isolating RNA for the study of small RNAs.

**Figure 3:**
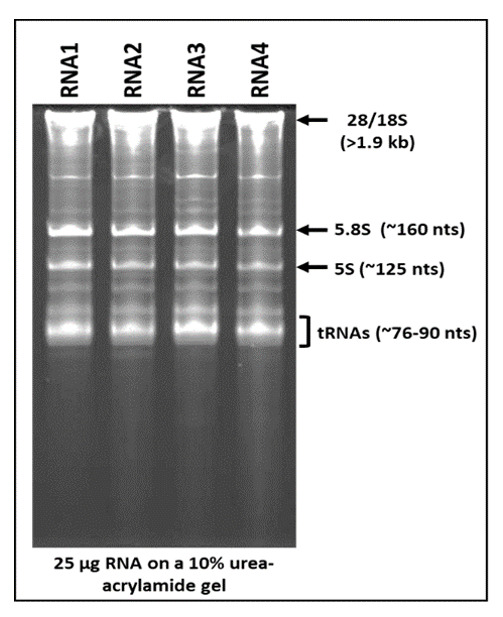
Analysis of RNA banding pattern and quality using acrylamide gels. TRIzol-extracted whole cell RNA (25 µg) from HC11 (RNA1 & 2) and 293T cells (RNA3 & 4) were electrophoresed on 10% 7M urea-acrylamide gels and stained with ethidium bromide. Identity of some of the RNA species is based on band size and intensity and is labelled on the side.

Next, we studied whole cell RNA upon electrophoresis on 10% 7 M urea-acrylamide gels to determine the banding pattern obtained, with a focus on the smaller RNA species upon ethidium bromide staining which have been less well studied. As can be seen in Figure 3, a ladder pattern of RNAs could be observed, suggesting isolation of good quality RNA, rather than a smooth smear which is more suggestive of degraded RNA [42]. The clearly stained RNA species started from the wells (representing large RNAs > 1.9 kb) to RNA clearly visible halfway down the gel as five prominent RNA species of which four could be identified as: 28 and 18S RNAs (>1900 nt in size) banding right below the wells, followed by 5.8S (~160 nt), 5S (~125 nt), and tRNAs banding between ~76–90 nt [38]. Several other RNA species banding in the range of 1.9 kb–90 nt could be observed, probably consisting of mRNAs (>500 nt in length), long non-coding RNAs (lncRNAs, 1–10 kbs, and other RNA transcripts). A light hint of smaller heterogenous RNA species indistinctly visible could also be observed (though barely) starting from immediately below the tRNA bands that went all the way down the gel, representing the RNA species smaller than 76 nt, presumably consisting of the pri- and pre- as well as mature forms of miRNAs.

### 3.1. Liquid Hybridization Is More Sensitive Than Northern Blotting for mRNAs

**Figure 4:**
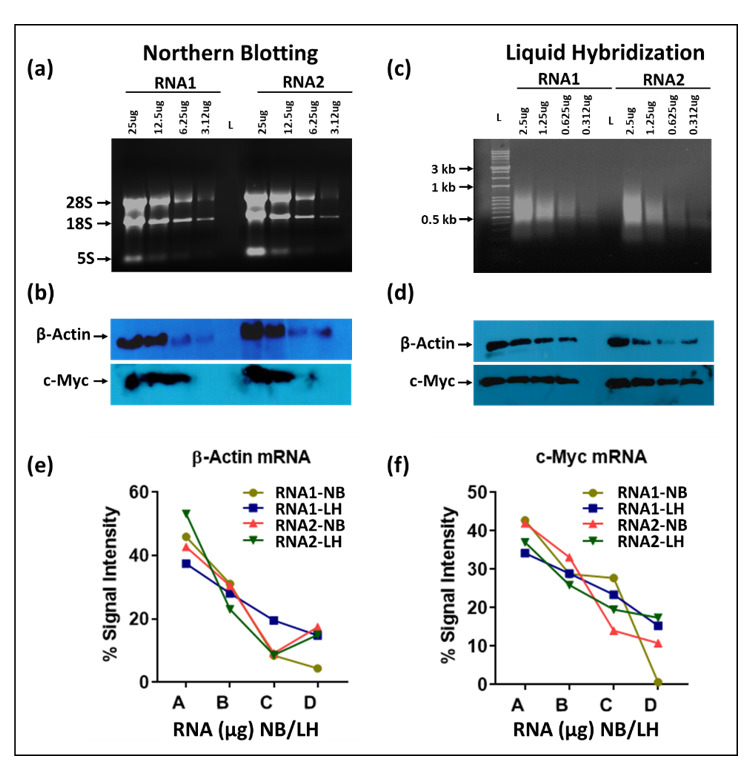
Comparative analysis of northern blots (NB) and liquid hybridization (LH) techniques. For northern blotting, serial dilutions of TRIzol-extracted RNA samples (25, 12.5, 6.25 and 3.125 μg each) were loaded onto a 1.2% denatured formaldehyde-agarose (FA) gels followed by semi-dry transfer to a nylon membrane and hybridization with biotinylated probes (50 pmol/mL). Whereas for liquid hybridization, serial dilutions of RNA samples (2.5, 1.25, 0.625 and 0.3125 μg each) were subjected to hybridization with the same amount of biotinylated probe (10 pmol/reaction) followed by Exonuclease I (Exo-I) treatment. After hybridization, the hybridized mixtures were loaded on non-denaturing agarose gels in MOPS buffer followed by transfer to a nylon membrane(s) using a semi-dry blot. After transfer, each membrane was UV crosslinked and EDC cross-linked for 2 hrs at 80 °C followed by incubation with HRP-conjugated streptavidin and detection using X-ray film. Ethidium bromide was added to the gels for visualization under UVP. (**a**) Gel image of ethidium-bromide-stained FA gels before transfer to a nylon membrane. (**b**) Northern blot showing expression levels of different mRNAs, including β-Actin and c-Myc after transfer to a nylon membrane and stripping. Images were taken using X-ray films. (**c**) UVP image of the hybridized RNA-probe mixture on a non-denaturing agarose gel before transfer. (**d**) Expression levels of selected mRNA(s) using X-ray film after transfer to nylon membranes. (**e**,**f**) Quantitative analysis of mRNAs expression levels after northern or liquid hybridization techniques. Image quantification was performed using GelQuant.NET software. Percent intensities were measured for each mRNA and plotted against the corresponding amount of RNA. X-axis labels: A = 25 or 2.5 μg; B = 12.5 or 1.25 μg; C = 6.25 or 0.625 μg; D = 3.125 or 0.3125 μg RNA).

Next, we compared the sensitivity of the liquid hybridization technique with northern blotting for the detection of mRNAs. For northern blotting, RNA was loaded as two-fold serial dilutions on 1.2% formaldehyde-agarose (FA) denaturing gels starting from 25 µg per well, along with ethidium bromide. Figure 4a shows the integrity of the RNA after electrophoresis which was followed by the northern blotting using an ~465 bp probe against β-Actin mRNA labeled at the 5′ end with biotin (Table 1). To develop the signal, the biotinylated probe was detected by incubation by a streptavidin linked to multiple molecules of horse radish peroxidase (poly-HRP) to enhance signal detection. This was followed by stripping and hybridization of the blot with an ~501 bp probe against c-Myc (Figure 4b and Table 1). As expected, a clear single band could be observed for each gene of the expected size in the X-ray image, decreasing steadily with the corresponding two-fold dilutions that were used for the two mRNAs used.

In case of liquid hybridization, we started with 10-fold less RNA, starting from 2.5 µg, and diluted it two-folds to 1.25, 0.625, and 0.3125 µg that were subjected to in-solution hybridization. Less RNA was used for the liquid hybridization since it is thought to be more sensitive than northern blotting [32,36,43,44]. The different dilutions of RNA were hybridized with the same amount of oligonucleotide version of biotinylated probes against the same mRNAs. Based on a cumulative analysis of current literation, hybridization was conducted in a unique manner where an initial denaturation step at 94 °C was followed by a stepwise decrease in temperature to allow successful annealing of the oligonucleotide probe to the target RNA, irrespective of its melting temperature (see Materials and Methods Section 2.3.1 for details). This was followed by treatment with Exonuclease I (Exo-I) enzyme to digest unhybridized probe (Figure 1), and the samples were loaded onto non-denaturing agarose gels, as described in Materials and Methods Section 2.3.2. Before transfer onto a nylon membrane, the gel images were taken using the UVP imaging system (Figure 4c). 

As can be seen, upon liquid hybridization and Exo-I treatment, the RNA now appeared as a smear as opposed to the distinct ribosomal bands that were apparent earlier. After transfer and signal development, clear single bands of decreasing intensity could be observed for both the β-Actin and c-Myc (Figure 4d). Quantitation of the bands after hybridization and signal development revealed that a similar trend of expression could be observed as that of the northern blotting technique. Densitometric analysis of the bands captured on X-ray film confirmed the similar levels of signal intensity and trend of expression between both groups (Figure 4e,f). This was despite having ten-fold less target RNA in liquid hybridized samples compared to the northern blot samples. Considering that in both cases, excess probe was used, these results reveal that the liquid hybridization is ~10-times more sensitive than the northern blotting technique and similar results could be attained even while using up to 10 times lesser amounts of RNA. This makes liquid hybridization an ideal and more sensitive technique for analyzing genes with lower expression levels. Furthermore, liquid hybridization allows one to investigate the expression of multiple targets simultaneously on a single membrane since once the hybridized samples are size fractionated and transferred to a blot, the blot is processed for sample detection and not hybridized with different probes, as is the case with northern blotting (Figure 1). However, the advantage of the northern blotting remains that it allows re-use of the nylon membrane with a new probe after a thorough stripping procedure, as shown in Figure 4b.

### 3.2. Liquid Hybridization for Small RNA Detection

**Figure 5:**
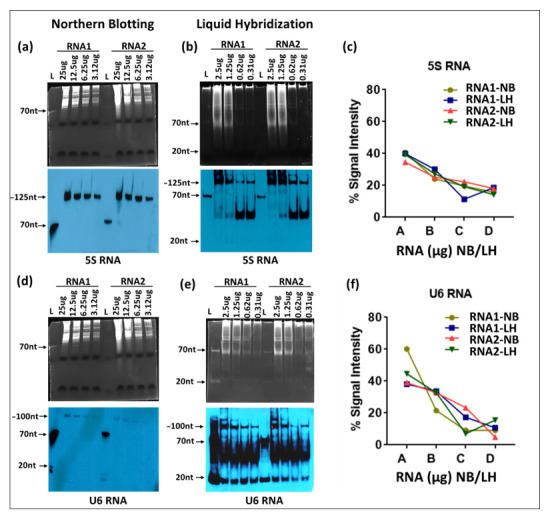
Liquid hybridization (LH) shows a similar trend for small RNA expression but with higher sensitivity and background when compared to northern blot (NB) analysis. For northern blotting, serial dilutions of TRIzol-extracted RNA (25, 12.5, 6.25 and 3.125 μg each) were loaded on 15% urea-acrylamide gels followed by semi-dry transfer to a nylon membrane and hybridization with biotinylated single-stranded oligonucleotide probes (50 pmol/mL). Whereas for liquid hybridization, serial dilutions of RNA (2.5, 1.25, 0.625 and 0.3125 μg each) were subjected to hybridization with biotinylated probe (10 pmol/reaction) followed by Exo-I treatment. After hybridization, the RNA/probe mixtures were loaded on 15% non-denaturing acrylamide-TBE gels followed by transfer onto nylon membranes using semi-dry blotting. Gels were stained with ethidium bromide for visualization under UVP, followed by semi-dry transfer onto nylon membrane. These were subjected to UV crosslinking and incubation with HRP-conjugated streptavidin before detection with X-ray film. (**a**) Gel image of extracted RNA from cell lines loaded in serial dilutions before transfer to a nylon membrane. After transfer, the membrane was probed with biotinylated 5S RNA small RNA, and expression was detected using X-ray film. (**b**) Gel image of the hybridized mixture of RNA with 5S RNA-biotinylated probe before transfer followed by detection using a UVP imaging system. (**c**) Quantitative analysis of 5S RNA expression levels after northern blot or liquid hybridization techniques. For 5S RNA, the ~125 nt band was used for quantitation purposes. X-axis labels: A = 25 or 2.5 μg; B = 12.5 or 1.25 μg; C = 6.25 or 0.625 μg; D = 3.125 or 0.3125 μg RNA). (**d**) Gel image of extracted RNA from cell lines loaded in serial dilutions before transfer to the nylon membrane. After transfer, the membrane was probed with biotinylated U6 small RNA probe and HRP-labelled streptavidin, and expression was detected using X-ray film. (**e**) Gel image of a hybridized mixture of RNA and U6 biotinylated probe before transfer. U6 expression was detected using X-ray film. (**f**) Quantitative analysis of U6 expression levels after northern or liquid hybridization techniques. X-axis labels: A = 25 or 2.5 μg; B = 12.5 or 1.25 μg; C = 6.25 or 0.625 μg; D = 3.125 or 0.3125 μg RNA). Image quantification was performed using GelQuant.NET software. Relative % intensities were measured for each mRNA and plotted against the corresponding amount of RNA.

Next, we compared northern blotting with the liquid hybridization technique in detecting small RNAs using the same sequentially diluted RNA samples. The RNA samples were electrophoresed on 15% denaturing 7M urea-acrylamide gels to allow detection of smaller sizes of RNAs and tested by transfer to nylon membranes for classical northern analysis. The membranes were hybridized with a biotinylated oligonucleotide probe against the small RNA, 5S RNA (Table 1), and the signal from the ~125 nt band was detected using the X-ray film detection system. The signals obtained were observed to be decreasing as per the RNA dilutions used in the experiment (Figure 5a). The used blot was stripped and re-hybridized with another small RNA probe against U6, followed by detection via X-ray film. Once again, the band intensities correlated with sequential dilution of RNA (Figure 5d). Moreover, as expected, the 5S RNA transcripts were much more abundant than the U6 RNA, as has been reported earlier where a vertebrate cell has ~7 × 10^6^ copies of 5S RNA per cell compared to only 4 × 10^5^ copies of U6 RNA [45].

For liquid hybridization, the RNA samples were hybridized with either the 5S or U6 DNA oligonucleotide probes end-labeled with biotin at the 5′ end, digested with Exo-I, and the hybridized samples were electrophoresed on non-denaturing 12% TBE acrylamide gels, followed by detection. As observed with the ethidium bromide-stained gels for liquid hybridized samples, the RNA samples for the small RNAs also showed a diffused pattern of staining, starting from the wells up to about halfway down the gel (Figure 5b). Upon transfer of the hybridized RNA followed by detection via X-ray films, liquid hybridized samples showed two specific bands, one that was specific for 5S RNA (~125 nt in length) and the other around 50 nt (Figure 5b). The decrease in band intensity of the 125 nt band correlated positively with RNA dilutions, but not that of the 50 nt band (Figure 5b). In fact, it was very faint for the higher concentrations of RNA samples and intense for the diluted samples. Based on the size and intensity of bands, we believe that this band represents primer/dimers that remained after liquid hybridization, less when the RNA was more and more when the RNA was less, as expected. Higher exposure of the blot revealed a band ~20 nt that was present in some of the lanes, but not others. The inconsistent presence of this band and its size suggests that this band represents residual probe that was either unhybridized or left undigested by the Exo-I enzyme.

Liquid hybridization for U6 small RNA after signal detection revealed three bands similar to the 5S blot. However, all three of these bands were quite strong: one band was specific for the U6 RNA (~100 nts long), one again ~50 nts long that we suspect represents primer/dimers, and the third one banding at ~20 nts, revealing the presence of unhybridized/undigested probe (Figure 5e). Both the primer/dimer and unhybridized/undigested probe bands for the U6 blot were a lot stronger than those observed for the 5S blot (Figure 5b), which probably is due to the lower concentration of the target RNA present in the samples. This observation confirms our conclusion that the band at ~50 nt represents primer/dimers. In terms of the specific signal, although both techniques showed similar trends of expression related to the amount of the RNA present in the sample, the northern blotting technique was much less sensitive than the liquid hybridization technique (Figure 5a,d vs. Figure 5b,e), confirming the observations made for the mRNAs. However, when one compares the relative mRNAs between 5S and U6, the northern technique was better at assessing the expected ratios of these RNAs than liquid hybridization that gave bands of nearly similar intensity for the two types of RNAs despite a 17.5-fold difference in their absolute amounts as well as probe excess [45]. The reason for this is probably the higher efficiency of oligonucleotide probes to form primer/dimers for the U6 RNA than the 5S RNA, perhaps due to differences in target RNA expression.

### 3.3. Biotinylated Primers Used as Probes in Liquid Hybridization Must Be Gel Purified

**Figure 6:**
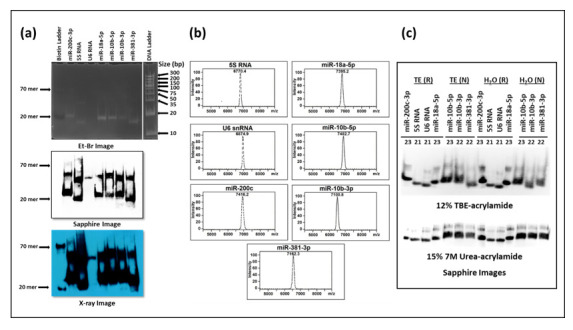
Biotinylation of oligonucleotide primers results in the formation of higher molecular weight species. (**a**) Analysis of the biotinylated primers on 12% TBE non-denaturing acrylamide gels stained with ethidium bromide (top panel) followed by their transfer onto nylon membranes, incubation with horse-radish peroxide (HRP)-linked streptavidin, and test of the HRP activity using luminol substrate. The chemiluminescent signal was detected either by the Sapphire image analyzer (middle panel) or X-ray development (bottom panel). (**b**) MALDI-TOF mass spectrometric analysis of the synthesized oligonucleotides provided by the manufacturer. (**c**) Test of biotinylated primers after heating (95 °C for 5 min followed by cooling on ice). The denatured oligos were analyzed on 12% TBE non-denaturing acrylamide gels (top panel) or 7 M urea/15% acrylamide denaturing gels followed by processing and signal detection as described in panel (**a**). Detection of the signal was by the Sapphire image analyzer. R = RNA loading buffer with formamide (denaturing); N = Native, non-denaturing loading buffer.

Next, we asked if we could get rid of the primer/dimers that were being observed in the liquid hybridization technique to improve visualization and ensure the results were not compromised. Increasing the temperature of hybridization or decreasing primer concentrations did not help remove the primer/dimer smears in the liquid hybridization reactions. To determine whether the primers being used could have been the problem, the biotinylated single-stranded oligonucleotides were analyzed on 12% acrylamide non-denaturing gels followed by their transfer onto nylon membranes, incubation with HRP-linked streptavidin, and test of the HRP activity using luminol substrate (Figure 6). The chemiluminescent signal was detected either by the Sapphire image analyzer or by developing the X-ray film. As can be seen from Figure 6a, two distinct bands could be observed that were clearly visible on the blot developed by either Sapphire (middle panel) or X-ray film (bottom panel) respectively: one corresponding to the expected size at ~22–24 nts and other one around 50–60 nts at ~ twice the size of the primers. This was surprising since the mass spectrometric analysis of the primers provided by the manufacturer revealed clean and tight single peaks according to size (Figure 6b), confirming the correct size and monomeric nature of the purchased oligonucleotides. Denaturing the primers at 95 °C and analyzing on non-denaturing 12% acrylamide gels or electrophoresing them under denaturation conditions (7M urea) did not get rid of the higher molecular weight species (Figure 6c top and bottom panels, respectively). This analysis confirmed that the higher molecular weight species was not due to the usual primer/dimers formed because of fortuitous self-base pairing that would have fallen apart under the denaturing conditions. Rather, they represented primers that were covalently bound to each other, probably a result of the biotinylation process, since these higher molecular weight species were not apparent in the mass spectrometric analysis conducted prior to biotinylation by the manufacturer (Figure 6b).

**Figure 7:**
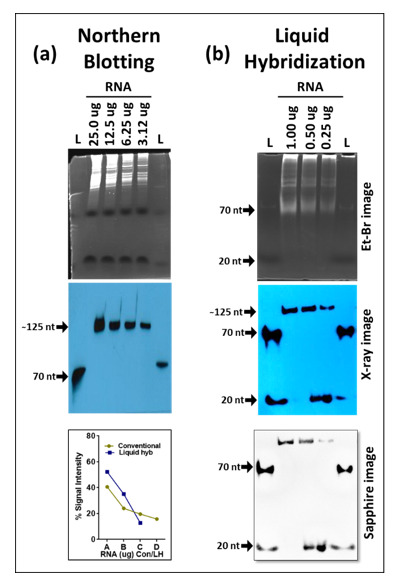
Liquid hybridization (LH) assay for the detection of 5S RNA after gel purification of biotinylated primers. (**a**) Northern blot (NB) for RNA1 (25, 12.5, 6.25 and 3.125 μg) from Figure 5a is shown again in comparison to results of the same RNA1 sample using liquid hybridization in panel (**b**). For northern blots, the samples were loaded on 15% urea-acrylamide gels followed by semi-dry transfer to a nylon membrane and hybridization with biotinylated single-stranded probes (50 pmol/mL). Whereas for liquid hybridization, serial dilutions of RNA (1.0, 0.5, and 0.25 µg each) were subjected to hybridization with 10 pm/reaction biotinylated probe that had been purified from acrylamide gel to remove the higher molecular weight species in the primer mixture. The hybridized samples were Exo-I treated and loaded on 15% non-denaturing acrylamide-TBE gels followed by transfer onto nylon membranes using semi-dry blotting. Gels were stained with ethidium bromide for visualization under UVP, followed by semi-dry transfer onto nylon membrane. These were subjected to UV crosslinking & baking and incubation with HRP-conjugated streptavidin before detection with X-ray film or via Sapphire gel imager. The graph shows quantitative analysis of 5S RNA expression levels after northern and liquid hybridization techniques. The ~125 nt band was used for quantitation purposes in both NB and LH blots. X-axis labels: A = 25 μg; B = 12.5 μg; C = 6.25 μg; D = 3.125 μg RNA) for NB, and A = 1 μg; B = 0.5 μg; C = 0.25 μg for LH. Image quantification was performed using GelQuant.NET software. Relative% intensities were measured for 5S RNA and plotted against the corresponding amount of RNA.

Based on these results, we extracted the correct size oligos from the acrylamide gels and used only those as probes in our liquid hybridization analysis for 5S RNA described earlier. As can be seen in Figure 7, the correct ~125 nt band could be easily detected that decreased according to dilution of the RNA sample. Once again, its intensity was similar to the intensity of the 5S band observable upon northern blotting despite using only 1 µg of RNA rather than 25 µg in the northern blot, confirming the ~ 10 times enhanced sensitivity of the liquid hybridization assay for small RNAs. The correct target band was present along with the ~20 nt band corresponding to undigested probe that remained after hybridization, less when the target RNA was more and more when the target RNA was diluted, as expected (Figure 7b More importantly, these bands were present in the complete absence of the primer/dimer species that was strongly noticeable before Figure 5b,e. Thus, purification of the biotinylated oligos on acrylamide gels allowed us to get rid of the higher molecular weight complex that we think is an artefact of the biotinylation process. Based on these results, we recommend that users test their biotinylated oligos for such artefacts on gels prior to use in liquid hybridization assays, and if present, the oligos should be gel purified to ensure proper results. Alternatively, gel purified biotinylated oligos should be purchased.

### 3.4. Liquid Hybridization Is More Sensitive Than Northern Blotting for miRNA Detection

**Figure 8:**
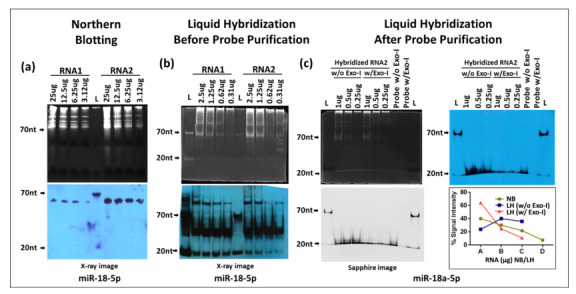
Liquid hybridization (LH) assay is more sensitive than northern blotting (NB) for miRNA detection. Comparative analysis of the sensitivity of northern and liquid hybridization techniques to detect miRNA expression before and after probe purification. (**a**) Northern blotting. Serial dilutions of TRIzol-extracted RNA1 & RNA 2 (25, 12.5, 6.25 and 3.125 μg each) were electrophoresed on 15% urea-acrylamide denatured gel followed by semi-dry transfer to a nylon membrane and hybridization with biotinylated oligos (500 pmol/10 mL). Liquid hybridization (**b**) before and (**c**) after probe purification. For all liquid hybridizations, serial dilutions of RNA samples (2.5, 1.25, 0.625 and 0.3125 μg each) were subjected to hybridization with the same amount of biotinylated oligos (10 pmol/reaction) followed by Exo-I treatment. The hybridized mixtures were loaded on 15% non-denatured acrylamide-TBE gel followed by transfer on a nylon membrane(s) using a semi-dry blotter. Gels were stained with ethidium bromide for visualization under UVP, followed by their transfer using a semi-dry blotter. After transfer, each nylon membrane was subjected to UV crosslinking and incubation with HRP-conjugated streptavidin before detection by X-ray and Sapphire gel imaging system. Panel c also shows quantitative analysis of expression levels of miR-18-5p after liquid hybridization northern technique. X-axis labels: A = 25 or 2.5 μg; B = 12.5 or 1.25 μg; C = 6.25 or 0.625 μg; D = 3.125 or 0.3125 μg RNA. Image quantification was performed using GelQuant.NET software. Relative % intensities were measured for each mRNA and plotted against the corresponding amount of RNA. For northern blotting, the precursor mRNA form was quantified since that was the only band visible, while for liquid hybridization, only the mature miRNA forms (~20 nt) were quantified.

Next, we compared the sensitivity of liquid hybridization with northern blotting for miRNAs for which it was originally developed [32,36]. For northern blotting, total cellular RNA samples starting from 25 µg and their two-fold dilutions were electrophoresed on denaturing 12% urea-TBE acrylamide gels, as described earlier for small RNAs, followed by detection of miR-18-5p using 5′-end labeled biotinylated oligonucleotide probes against the mature form of the miRNA (Figure 8a and Table 1). The 12% gel was used since miRNAs undergo maturation from pri- and pre-forms that can range from 1000 nts to ~60–125 nts with the final mature form being around 20–25 nts [1,30,46]. As can be seen, only a band of ~60 nt size was detectable in the two RNA samples (RNA1 and RNA2) according to RNA concentrations, with no detection of the mature form that should have appeared around 20–25 nts (Figure 8a). In contrast, liquid hybridization was able to detect the short ~20 nt mature form of miR-18-5p (Figure 8b) as well as a band > 100 nt. Additionally, a complex could be observed in between the 70 and 20 nt size with a distinct band around 50 nts (Figure 8b), similar to what was observed for the small RNAs under liquid hybridization conditions (Figure 5) that was attributed to primer/dimer formation. To confirm this possibility, the biotinylated oligonucleotide probe was purified via acrylamide gel electrophoresis and used in the same assay again. As can be seen in Figure 8c,d, gel purification of the probe led to a complete disappearance of the >100 nt as well as the ~50 nt band attributed to non-specific binding and primer/dimer formation, leaving only the ~20 nt size band visible, confirming our earlier observations that the biotinylated oligonucleotide primers must be gel-purified prior to use in these assays. Interestingly, we have made this observation not only for biotinylated oligos commercially purchased but also with oligos that were biotinylated by ourselves using a kit (data not shown), suggesting that this artefact is due to the biotinylation process, irrespective of whether the oligos were biotinylated commercially or self-biotinylated using a kit.

The liquid hybridization for miR-18-5p was performed both in the presence or absence of Exo-I so that one could distinguish between the signal coming from the probe bound to the target miRNA versus signal from unhybridized probe alone that would have remained single due to the excess concentration used. As can be observed, the dilution effect of the RNA was apparent in the samples with Exo-I, but not in samples without Exo-I, an effect which could be quantitated (Figure 8c). Together, these data show that miR-18-5p could be detected using the liquid hybridization method, while it could not be detectable using the northern technique under the conditions used. This is despite the fact that the northern blot was performed starting with 25 µg RNA versus liquid hybridization that was conducted using RNA starting with only 1 µg and the signal was still detectable at 0.25 µg (Figure 8c). Thus, liquid hybridization has at least 100-fold higher sensitivity of detection of miRNAs compared to northerns.

These results raise the question that if liquid hybridization is so sensitive, why was it unable to detect the ~60 nt pre-miRNA band that could be effectively detected upon northern blotting (Figure 8a). This band was not visible upon liquid hybridization in the absence of Exo-I, a condition under which we should have been able to detect not only the mature, but also the pri- and pre-forms of miRNAs. This observation suggests that the 60-nt band reflects a non-specific band and does not represent the pre- form of miR-18-5p, as suspected originally. Thus, it is probably an artefact of the use of only 21–23-nt sized oligonucleotide as a probe for detection purposes which could have fortuitous complementarity to some other expressed sequence in the genome. This makes the liquid hybridization technique not only a highly sensitive, but a highly specific technique to detect miRNAs.

In order to confirm this point, the northern blot analysis was repeated for miR-18-5p after probe purification to determine whether the ~60 nt form could be detected using this technique. As suspected, we were unable to detect any band on the northern blot for miR-18-5p after probe purification (data not shown). This observation suggests that: (1) the 60 nt band being observed earlier in miR-18-5p northern was an artefact of the probe primer/dimer prior to its purification, and (2) that northern blot is unable to detect miRNA expression under the conditions used in this study for either the pri-, pre-, or mature forms of miRNAs, while liquid hybridization can detect the mature form.

**Figure 9:**
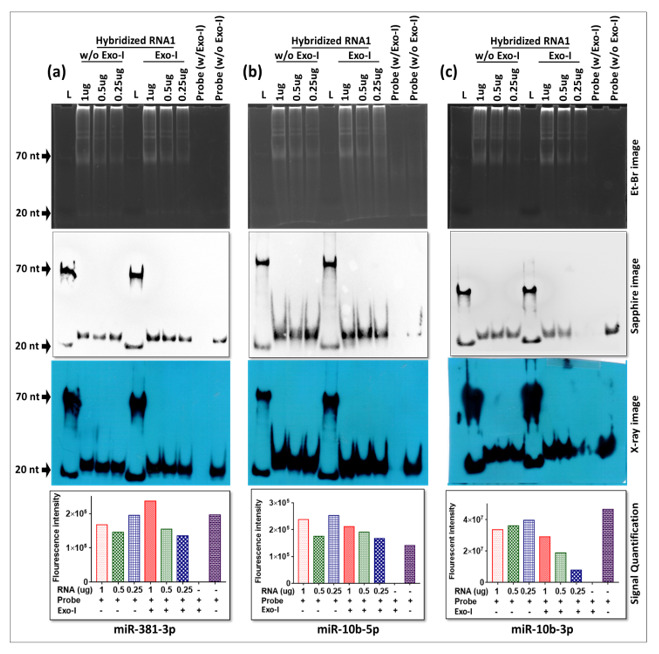
Successful detection of three miRNAs using liquid hybridization after probe purification. The assay was used to detect expression of: (**a**) miR-381, (**b**) miR-10b-5p, and (**c**) miR-10b-3p. Serial dilutions of RNA (1.00, 0.50 and 0.25 μg each) were subjected to hybridization with the same amount of gel purified biotinylated oligos (2.5 pmol/reaction). The hybridized mixtures either without (*w*/*o*) or with (w) Exo-I treatment were loaded on 15% non-denatured acrylamide-TBE gel followed by transfer on a nylon membrane(s) using a semi-dry blotter. Gels were stained with ethidium bromide for RNA visualization under UVP. After transfer, each nylon membrane was subjected to UV crosslinking and incubation with HRP-conjugated streptavidin before detection by the Sapphire imaging system or X-ray. After transfer, membranes were probed with pre-biotinylated miR-381-3p, miR-10b-5p and miR-10-3p probes, respectively, and expression of these miRNAs was detected using X-ray. Quantitative analysis of expression levels of miRNAs was performed using GelQuant.NET software. Relative % intensities were measured for each mRNA and plotted against the corresponding amount of RNA.

To confirm these observations, liquid hybridization was performed for the detection of three additional miRNAs, miR-381-3p, miR-10b-5p, and miR-10b-3p, where gel-purified biotinylated oligonucleotides were used as probes to determine if they could be successfully detected. As can be seen from Figure 9, only a single ~20 nt band could be observed in these blots, either in the absence or presence of Exo-I; however, a dilution effect of the RNA could be observed only upon the presence of Exo-I (see the histograms below the X-ray images). This reveals that liquid hybridization was 100% effective in successfully detecting the true signal from the three different miRNAs. Furthermore, in case of each miRNA tested, efficient miRNA signal could easily be observed till the last dilution tested (0.25 µg of RNA), confirming our earlier observation that in the case of miR-18-5p (Figure 8c) that our conditions of liquid hybridization are at least 10–100× more sensitive than the classical northern blotting using the same probes (data not shown).

**Figure 10:**
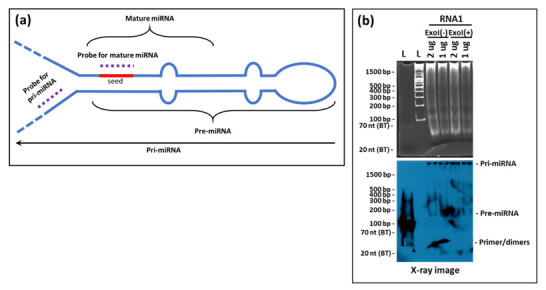
Successful detection of pri- and pre-miRNAs using a probe in the pri-/pre-miRNA region of miR-17-92. (**a**) Schematic representation of miRNA structure highlighting its mature, pre-, and pri-miRNA regions and probe locations. Figure adapted from [47]. (**b**) Liquid hybridization of HC11 RNA (RNA1) with a probe outside the seed sequence (within the pri/pre-miRNA region) with (+) or without (−) Exo-I. The samples were run on a 6% non-denaturing acrylamide gel. The two bands at the bottom in the Exo-I (−) lanes are biotinylated primers that form primer/dimers in the absence of Exo-I and are degraded in its presence (Exo-I (+) lanes).

Our assay was unable to detect the precursor forms of the tested miRNAs (the pri- and pre-forms) despite the high sensitivity of the liquid hybridization assay. This probably is due to the location of the probes used that encompasses the “seed” region of the miRNAs (Figure 10a). This region of miRNAs is involved in tight hairpin conformations in the target miRNAs and the probability of their reforming the hairpins under the liquid hybridization conditions are a lot more than binding to the probe (Figure 10a). Thus, the chances of detecting the precursor forms of miRNAs are miniscule with probes against the seed sequence. Instead, a better design would be to target the pri- region of the miRNAs which is found in a non-base-paired state. We have tested this possibility and our preliminary results show that indeed the probe in the pri/pre-miRNA region is able to detect the pri- and pre- forms of the miRNAs (Figure 10b), making liquid hybridization a powerful technique to study miRNA biogenesis as well.

### 3.5. Slot Blot Could Be an Alternative to Acrylamide Gel for miRNA Semi-Quantitation in Liquid Hybridization Assays

**Figure 11:**
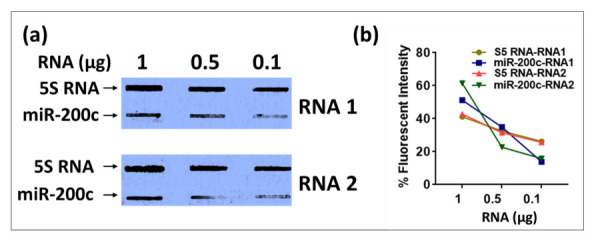
Liquid hybridization on the immobilized membrane using slot blot could be an alternative to acrylamide gel. Different concentrations of RNA1 and RNA2 (0.1, 0.5 and 1 ug, respectively) were used for liquid hybridization using 10 pmol of biotinylated RN5S or miR-200c probes per RNA concentration. (**a**) After overnight Exo-I treatment, hybridized mixtures were transferred to positively charged nylon membranes using slot blot, as per manufacturer instructions. After transfer, the UV-crosslinked membrane was incubated with HRP-conjugated streptavidin followed by 5S RNA or miR-200c signal detection using an X-ray. (**b**) Quantitative analysis of 5S RNA and miR-200c expression levels after X-ray detection. Image quantification was performed using GelQuant.NET software. Relative % intensities were measured for each RNA and plotted against the corresponding amount of RNA.

Finally, we asked whether liquid hybridization could be coupled with slot blotting for better quantitation purposes since only one species of mRNAs, small RNAs or miRNAs could be detected using this technique rather than their pre-forms. In this approach, the products of liquid hybridization were immobilized onto membrane and UV cross-linked rather than size fractionated, saving time and allowing better quantitation (Figure 11). Thus, instead of electrophoresing the liquid hybridization products on an acrylamide gel, the slot blotting technique was used to analyze the expression of two RNAs, 5S RNA and miR-200c, with sequential RNA dilutions in liquid hybridization reactions. After overnight Exo-I treatment, the samples were transferred onto nylon membranes using the slot blotting technique. The resultant blot was incubated with HRP-conjugated streptavidin and detected using X-rays film. Slot blot analysis of the liquid hybridized products showed a reduction of chemiluminescent signal correlating with the amount of RNA used, confirming that it could be used for semi-quantitation purposes (Figure 11a,b). Unlike samples run on acrylamide gel after the liquid hybridization, which can cause smeary bands more difficult to quantitate (Figure 5, Figure 8 and Figure 9), the slot blot for obvious reasons provides a single tight band for each of the tested samples, making it much easier to quantitate digitally (Figure 11b). Of course, the drawback of this approach is that slot blot is unable to distinguish amongst the different spliced forms of miRNA, but then, it allows faster quantitation of the mature form of the miRNAs for a quick assessment of their expression. 

### 3.6. Machine Imaging Reveals Inability to Detect ECL Signal in the Presence of Ethidium Bromide When Compared to X-ray Imaging for HRP-Conjugated Streptavidin-Biotin-Labeled Probes

The biotin-labeled product is visualized using the chemiluminescent detection kit. For detection, the membrane containing the biotinylated products are incubated with streptavidin protein conjugated with horseradish peroxidase (HRP). HRP provides enzymatic activity for detection using the substrate system like luminol (ECL). Upon enzymatic reaction, luminol results in chemiluminescence that can be captured either by the classical X-ray imaging or by the more modern imaging systems. In this study, we compared the signal detection sensitivity of both X-ray and the widely used imaging system, Typhoon (FLA9500). The Typhoon machine has prescribed wavelengths and filters for specific dyes that can selected. To detect HRP-luminol, the ECL wavelength was selected whereas, for ethidium brome, the EtBr wavelength was employed. In northern blotting, a stringent quality check is routinely performed before transfer to positively charged membranes. To check for RNA quality and integrity before transfer, agarose or acrylamide gels are stained with EtBr and viewed either by UVP gel imaging system or by Typhoon imager using the EtBr filter.

**Figure 12:**
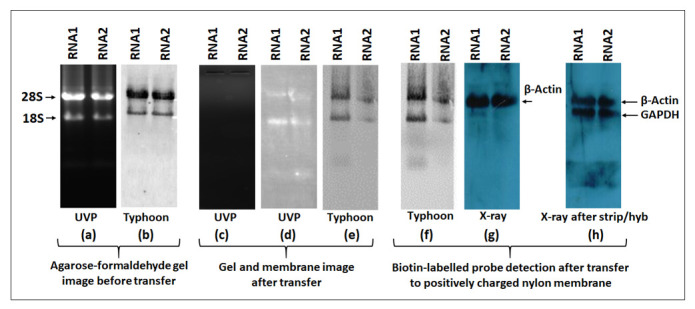
Inability of Typhoon imaging to detect the ECL signal compared to X-ray imaging. RNA1 and RNA2 were subjected to electrophoresis on 1.2% denatured agarose-formaldehyde gel. (**a**) The gel image was taken with a UVP gel doc system after ethidium bromide (EtBr) staining, but before transfer to positively charged nylon membranes. (**b**) The gel image was taken with the Typhoon imaging system before transfer to positively charged nylon membranes. (**c**) The gel image was taken after transfer to a nylon membrane by UVP gel doc. (**d**) The nylon membrane image was taken by UVP after transfer. (**e**) The nylon membrane image was taken by Typhoon after transfer using the EtBr filter. (**f**) Detection of a biotin-labeled probe by Typhoon using ECL filter. (**g**) Detection of β-actin biotin-labeled probe by X-ray. (**h**) Detection of GAPDH biotinylated probe by X-ray after mild stripping of the blot shown in panel (**g**) after hybridization with β-actin-biotinylated probe.

Figure 12a shows gel imaging before the transfer stage. Both systems showed intact 28S and 18S bands and confirmed the quality and integrity of the RNA. After transfer, the gel was once again viewed under UVP that confirmed the complete transfer of RNA from the gel to the nylon membrane (Figure 12c). After transfer and UV-crosslinking, the nylon membrane was viewed under both UVP and Typhoon using the EtBr filter. Both systems confirmed the presence of RNA on the nylon membrane (Figure 12d,e). The membrane was then hybridized overnight with a β-actin PCR probe labeled with biotin followed by blocking and incubation with HRP-conjugated streptavidin. The Pierce ECL plus substrate was used to detect the chemiluminescent signal either by Typhoon or X-ray. Using the ECL plus filter in Typhoon, we detected two bands (Figure 12f) that were present in the same position as 28 and 18S RNAs detected under the EtBr filter. However, X-ray imaging showed a single distinct band of β-actin (~2.2 kb), the true hybridization signal that was not observed when imaged using Typhoon. To confirm these results, we mildly stripped the membrane and hybridized the blot with another probe, GAPDH. Once again, the GAPDH band (~1.8 kb) could only be detected using X-ray detection and not by the Typhoon imager. We observed a similar, persistent trend throughout our study that suggest that caution must be used when using the Typhoon imager for this purpose since it may not be able to detect biotin-streptavidin-HRP signals in the presence of ethidium bromide-stained RNA.

## 4. Discussion

Northern blot is considered a gold standard to determine RNA transcript form and expression semi-quantitatively. Despite this, several caveats make northern blotting unpopular amongst modern molecular biologists. Unlike other techniques like western or Southern blotting, northern analysis requires huge amounts of RNA that is labile and highly sensitive to degradation when compared to DNA or proteins. Northern blotting is also a time-consuming protocol and detection of small RNAs could take from three days up to a week or more, depending upon how many transcripts need to be analyzed. Moreover, northern shows the least sensitivity when compared to other techniques like microarrays, RT-PCR and ribonuclease protection assays [23,24,25]. Other shortcomings of the technique include the use of radioisotope to make it sensitive, which by itself is considered a health hazard with special requirements for isotope handling and its disposal. Recent developments in biotechnology have allowed several modifications made to the basic northern protocol that has given researchers variants of northern blotting that are not only safer, but also quite sensitive. These modifications include the use of non-radioactive labels, such as digoxigenin (DIG) or biotin-labeled probes, chemical crosslinking to improve RNA attachment to membranes, and the use of RNA enrichment techniques [35,48,49]. However, northern blots have their own advantages that should be kept in mind, including the fact that they are a lot more straightforward, have less artefacts, and can provide proper size information of the transcripts [23]. They can also detect different sizes of transcripts present for the same gene, thus allowing study of alternative splicing or those studying RNA biogenesis, whether it is mRNA or miRNA [23].

With the discovery of miRNAs, liquid hybridization has been introduced as a possible alternative to classical northern blotting since miRNAs are difficult to detect due to their small size and low levels [32,36,43]. This method involves pre-hybridization of target RNA with oligonucleotide probes in a buffer followed by Exo-1 digestion to remove unhybridized probe and non-denaturing gel electrophoresis. Liquid hybridization allows the detection of miRNA using even non-radiolabeled probes [32,36,43,50]. However, these studies have had their own shortcoming, making data interpretation at times difficult, including the need of showing full gel data rather than specific bands and the use of molecular weight ladders to show the approximate size of bands after hybridization [32,36,43,44]. Furthermore, although these studies have mentioned the use of Exo-I to digest excess, non-hybridized probe, no data is presented on results without Exo-I. This is important since partial Exo-I digestion could lead to false positive expression. Finally, some studies have performed liquid hybridization using slot or dot blots to detect signals [43,50]. However, as mentioned, one needs to keep in mind that this could lead to misinterpretation of quantitation of mature miRNAs in case pri- and pre-miRNAs are present.

### 4.1. Development of Highly Sensitive Liquid Hybridization Technique for mRNA, Small RNA and miRNA Detection

In this study, we developed non-radiolabeled, biotin-based-northern blots as well as highly sensitive liquid hybridization assays and systematically compared them for all three sizes of RNAs: mRNAs, small RNAs, and miRNAs. The liquid hybridization technique was taken several steps forward by not only using it to detect miRNAs via the biotin-streptavidin detection system, but extending it to the study of small RNAs and mRNAs, an aspect that has not been reported before to the best of our knowledge, making our study more versatile and broader than reported previously. Our work further highlights several technical aspects of the technique that have not been addressed before, including: (1) introduction of a pan-hybridization step that allows probes with different melting temperatures to be hybridized using one program, (2) use of poly-HRP to enhance signal, (3) demonstration of artefacts of probe biotinylation that necessitate their gel purification, (4) test of RNA samples in the presence and absence of Exo-I to confirm target specificity and allow study of RNA biogenesis, (5) use of EDC to enhance cross-linking for northern blotting, and (6) uncovering caveats of modern image analyzers for the detection of luminol signals in the presence of ethidium bromide, etc. Thus, the incorporation of these technical aspects helped us develop more sensitive, efficient, cheaper, and faster liquid hybridization assays (as well as northern blots) for RNA detection.

For example, incorporation of improvised hybridization conditions for liquid hybridization assays based on published studies [32,36,43,44,50] avoids pre-optimization steps and further shortens the duration of the technique. The hybridization step was carried in a graded manner, allowing efficient annealing of the probe to the target RNA irrespective of the melting temperature of the oligonucleotide probe. This allowed the use of only one hybridization program for oligonucleotide probes with different melting temperatures using any temperature within a specific range. Using such an approach provided the same sensitivity as that of the optimized hybridization temperature (data not shown). Similarly, we observed that chemical crosslinking via EDC (which was proposed as an alternative to baking for miRNA detection in northern blotting [33]) enhanced signals after UV crosslinking for northern blotting, but had a similar effect on liquid hybridized samples whether they were crosslinked by UV light followed by baking or EDC crosslinking, obviating the need of EDC in liquid hybridization assays. EDC is thought to mediate RNA immobilization via interaction between the 5′ terminal phosphate of the nucleic acid and the amine group of nitrocellulose and/or nylon membranes. This leaves the immobilized nucleotide bases receptive to complementary probes during hybridization, thus increasing sensitivity of the assay [33]. Therefore, we recommend EDC crosslinking only for northern blots after UV cross-linking to enhance results. Thus, our assays are not dependent upon radioactivity or specialized reagents like LNA nucleic acids or DIG labels, and can be conducted by any laboratory that has access to routine molecular biology equipment and reagents.

Compared to northern blotting, we found liquid hybridization technique to have a much shorter procedure that could be completed in essentially a day versus 3–5 days or more for northern blotting, especially in cases of re-use of blots for multiple transcripts. In this study, we used overnight Exo-I digestions to ensure complete digestion, a step that can easily be reduced to 30 min by increasing enzyme concentrations (data not shown). We opted for low exonuclease concentrations for the overnight time periods to reduce confounding factors (discussed below) as well as ease of work since the experiment could be set up in the evening and easily completed the next day. We also observed liquid hybridization to be 10—100 folds more sensitive than the classical northern blotting when using biotinylated probes for the detection of mRNAs, small RNAs, and miRNAs (Figure 4, Figure 7, Figure 8 and Figure 9). Finally, compared to northern blotting which allows assessing the relative level of gene expression between different genes, we found that liquid hybridization can only compare expression levels between the same gene. In other words, liquid hybridization allows the study of expression differences only within one gene and not across multiple genes since similar to PCR, each probe has its own efficiency of hybridization to the target RNA and the two techniques should be used according to the needs. Thus, liquid hybridization can be used to compare between the same miRNAs (for example), but it cannot be used to estimate the relative levels of one miRNA from another. However, the use of reference controls could allow one to overcome this caveat.

### 4.2. Strength of Our Assays Compared to Published Results

The Supplementary Table S1 provides a comparative analysis of the present study with some other studies that have either used northern blotting or liquid hybridization assays to study miRNAs [32,35,36,42,48,51]. It notes important details of each study, such as the target RNAs studied, RNA amounts used, assay sensitivity, types of probes and their amounts, the crosslinking method used, enzymes used for detection, detection method and choice of substrate, detection details and exposure times. As can be seen, it allows one to compare across studies, determine which study used which type of starting RNA material, probes, and detection methods, and reveals the relative sensitivity of assays developed by each study. The strength of our study is that our liquid hybridization assays used existing techniques and biotinylated DNA probes, yet we were able to improve the sensitivity of miRNA detection down to the level of radioactive and/or LNA probes without enriching for small RNAs which is quite remarkable [compare amounts of RNA and probes used and the level of sensitivity observed in the Supplementary Table S1 between our liquid hybridization assay and those of Kim et al. [35] and Li et al. [50]. Thus, even at total RNA concentrations of 250 ng, we could easily detect various miRNAs with 10 pmol probe/reaction (Figure 8 and Figure 9). Unfortunately, our northern blot was not able to detect miRNAs; however, in terms of sensitivity for mRNA and small RNA detection, we could easily dilute the total RNA down to ~3 µg and still detect signal, a starting amount which is standard or in fact a bit at the lower end of northern blots using radioactive probes. Furthermore, we could improve this level of sensitivity 10 times more using our liquid hybridization approach where we could go down to ~ 300 ng and still get even better signal than 3 µg total RNA in northern blotting (Figure 4 and Figure 5). These analyses reveal that our biotinylated northern blots are comparable to classical northerns for mRNA and small RNA detection, while our liquid hybridization assays are much more sensitive than blot based northerns for all three types of RNA detection. Furthermore, by using probes in the pri/pre-miRNA regions, one can detect not only the mature, but also the pri- and/or pre- forms of the miRNAs using the liquid hybridization technique (Figure 10).

### 4.3. Potential Role of Exo-I Treatment in Liquid Hybridization

**Figure 13:**
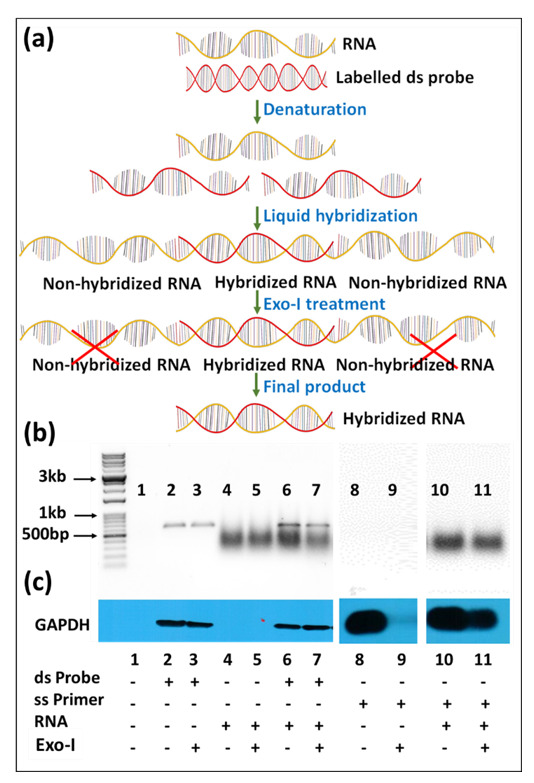
Exonuclease I (Exo-I) efficiently remove single-stranded oligos after liquid hybridization. Extracted RNA1 was hybridized with either biotin-labeled GAPDH double-stranded (ds) probe (lanes 1–7) or single-stranded biotinylated primer probe (lanes 8–11) using the liquid hybridization method. After completion of hybridization, the samples were divided into half and either treated with Exo-I overnight at 37 °C or left at 4 °C without Exo-I. Both the Exo-I-treated and untreated hybridized samples were electrophoresed on 1.2% non-denaturing agarose gels in 1× MOPS buffer without formaldehyde followed by transfer onto positively-charged nylon membranes using the semi-dry method. (**a**) Schematic diagram showing hybridization of RNA and double-stranded labeled probe in the liquid hybridization method. Treatment with Exo-I resulted in the same size of the product after hybridization. (**b**) Agarose gel image after ethidium bromide staining of the hybridized samples taken by the Typhoon imaging system before transfer to a nylon membrane. (**c**) X-ray detection of biotin-labeled probe/ product after transfer.

As mentioned above, for mRNA detection, liquid hybridization showed excellent sensitivity and was able to detect signals in 10 times less RNA when compared to the classical northern (Figure 4). Unlike small RNA or miRNA, the mRNA probe used was double-stranded prepared via PCR amplification using pre-biotinylated primers. In classical northern blotting, prior to hybridization, RNA is sized fractionated using gel electrophoresis and the resultant expressed mRNA is not dependent on the labeled probe size. This is not the case with liquid hybridization where, after the hybridization stage, Exo-I treatment was observed to remove un-hybridized single-stranded RNA (Figure 13a), in addition to excess probe when used as a single stranded primer [4,13]. This results in the same size of the hybridized product as the probe used (Figure 13a). To explore how Exo-I treatment functions, RNA was hybridized with either biotin-labeled double-stranded probe or single-stranded primers followed by overnight treatment of half the sample with Exo-I, while the other half remained untreated. The RNA samples hybridized with double standard probes showed a little bit decrease in signal of both input RNA and probe upon Exo-I digestion, as observed upon ethidium staining (Figure 13b, lane 6 vs. 7), suggesting that it was removing unhybridized single-stranded RNA in addition to excess probe. However, not much change in expression was observed either with or without Exo-I treatment upon image detection, revealing that dilution of sample RNA was necessary to ensure real signal was being observed when using double-stranded PCR probes for mRNA detection (Figure 13b,c, lanes 6 vs. 7). However, Exo-I treatment of RNA hybridized with biotinylated, single-stranded primers resulted in significantly decreased signal (Figure 13b,c, lanes 10 vs. 11). This is because as suspected, Exo-1 effectively removed the excess single-stranded primers from the reaction. These results show that there needs to be more controls in liquid hybridization assays to minimize false-positive signals of the labeled probes or primers.

Among the three classes of RNAs tested, liquid hybridization was most efficient at sensitively detecting miRNA expression. In fact, liquid hybridization was able to detect mature form of miRNAs undetectable even using 25 times higher amounts of total RNA in the classical northern blotting technique (Figure 8 and Figure 9). This lower sensitivity of the northern blotting for miRNAs could potentially be increased by the use of other labels, such as DIG combined with LNA probes [34,35], but we have not tested that. In case of mRNA detection, to avoid false-positive signals, we had to not only use Exo-I digestion, but also serially dilute the RNAs to differentiate between excess probe from the real signal. This was achieved by hybridizing the same amount of probe with sequentially diluted RNA followed by Exo-I treatment. This revealed a relative change in the output signal dependent upon RNA concentration, confirming that the output expression was due to the probe hybridized with the RNA and not from the double-stranded probe (Figure 4). Thus, for liquid hybridization for mRNA, the RNA sample should be tested in multiple dilutions along with their Exo-I digestion to ensure that the signal being observed is from the target RNA and not the double-stranded probe. For the small and miRNAs, we only used single-stranded oligo probes and their use necessitated the use of Exo-I to differentiate between the excess, unhybridized probe and hybridized RNA. In the latter case of miRNAs, RNA dilutions were tested, but were not necessary for the differentiation between the real and false signals.

We were also able to detect the same sensitivity of liquid hybridized signals after transferring the liquid hybridized-Exo-I-digested small RNA or miRNA samples directly on to the positively charged nylon membranes using slot-blot (Figure 11). This method can reduce liquid hybridization time effectively, but presents some limitations such as the inability to tell the size of the transcript. Thus, this modification of the technique should only allow detection of a single signal for all forms of the RNAs. This could result in an incorrect interpretation of results if one is suspecting that mRNA/miRNA processing may be affected in those samples. Thus, a prior test is needed to confirm that the RNA processing is not affected by before choosing liquid hybridization. Otherwise, this procedure should be quite accurate.

### 4.4. Pre-Purification of Biotinylated Primers Is Required for Better Results

One important observation in our study was that primer probes that are biotinylated must be gel-purified prior to their use in liquid hybridization assays if multiple bands are observed other than the expected one upon their gel electrophoresis (Figure 6). This observation was made after painstakingly not being able to get rid of the spurious primer/dimer bands that were being observed in our liquid hybridization assays for small RNAs and miRNAs (Figure 5 and Figure 8). These primer/dimers seemed to originate not from self-hybridization of the biotinylated primers being used since heating the probes at 95 °C did not get eliminate them or electrophoresing them under denaturing conditions of 7M urea (Figure 6). Rather, we suspect that there are biotin-biotin interactions taking place during the labeling process that irreversibly crosslinks the primers to each other, creating covalently stable dimers that must be removed via gel purification before the use of the biotinylated primers in liquid hybridization reactions. The evidence for this can be observed by the fact that, upon MALDI-TOF analysis of the commercially synthesized primers for size, only a tight peak of one size was observed (Figure 6b), which shows that the primer dimerization must have taken place post biotinylation of the primer. These linked primers probably have the capacity to bind to target RNA since they are still not base-paired, but the products would be of a different size due to the cross-linked nature of the primers, leading to the types of smears and non-specific bands that were observable in Figure 5 and Figure 8. 

### 4.5. Detection of False-Positive Signals after Using Machine-Based Detection

Another important observation in our study was the detection of false-positive signals after using machine-based detection of the HRP-catalyzed chemiluminescent signal using an ECL detection kit. After hybridization, mRNA, small RNA, or miRNA expression can be detected using the biotin-streptavidin-HRP conjugation method. X-ray has been traditionally used for the detection of HRP-luminol [52]. HRP oxidizes its chemiluminescent substrate luminol in the presence of peroxide and emits light at 425 nm that can be detected using an X-ray film [52]. Nowadays, machine-based laser scanners are replacing the traditional X-ray technique and are being widely used for the detection of nucleic acids and proteins. They are fast, detect multiple probe types and do not require special dark rooms, as required when using the X-ray method. In this study, we also used Typhoon biomolecular imager to detect mRNA, small RNA, and miRNA expression after incubating the hybridized membranes with HRP-conjugated streptavidin. We found that upon selecting the ECL-plus filter, the resultant signals detected with Typhoon were not the HRP-luminol signals, but actually EtBr signals (Figure 12). A similar observation was made when using the Sapphire biomolecular imager (data not shown). These results were surprising since we routinely use both the Typhoon and Sapphire biomolecular imagers for western blotting using the same HRP-luminol method. Our investigations revealed that these false signals were due to the presence of EtBr in the samples that have maximum emission at 590 nm (Figure 12). Based on these observations, we recommend the X-ray method for the detection of HRP-ECL signal to avoid detecting false signals emanating from the use of EtBr in northern blotting. Alternatively, one must either use other non-EtBr-based stains for visualizing RNA on gels or completely remove EtBr from the electrophoresed RNA samples prior to transfer to the membrane to avoid EtBr interfering with further downstream detection of the chemiluminescent signals. For liquid hybridization signal detection, Sapphire provided good results but with lower sensitivity, while Typhoon demonstrated slightly higher sensitivity but with bad resolution (compare results from the two imagers in Figure 6 and Figure 9 for example).

## 5. Conclusions

In summary, in this study, we developed and compared non-radiolabeled, sensitive, rapid, and cost-effective northern blots and liquid hybridization assays for mRNAs, small RNAs, and miRNAs that require minimum specialized reagents. Overall, we observed the sensitivity of our biotin-based northerns comparable to traditional northerns for mRNAs and small RNAs, while our liquid hybridization assays were even better and demonstrated sensitivity (>10–100 folds) for the detection of all three classes of RNAs. Unlike northern blotting, liquid hybridization was able to efficiently detect mature miRNAs using biotin-labeled probes and total RNA. Moreover, the traditional X-ray film method to detect HRP-luminol showed superiority over advanced laser machine-based technology. Overall, our study provides an effective experimental framework for investigators researching different RNA molecules and their functions at the molecular level in an economical and efficient manner.

## Figures and Tables

**Table 1:** List of primers used in this study.

Sr No	Target	Primer Sequence	Description
1	GAPDH	CAT GTT TGT GAT GGG TGT GAA CCA- (Forward (F))	580 bp probe
		GTT GCT GTA GCC GTA TTC ATT GTC- (−Reverse (R))	
2	β-Actin	CTT CTT TGC AGC TCC TTC G- (F)	465 bp probe
		ACA GCC TGG ATG GCT ACG- (R)	
3	c-Myc	CTG CGA CTG ACC CAA CAT CAG C- (F)	501 bp probe
		CGT AGC GAC CGC AAC ATA GG- (R)	
4	5S RNA	AGC CTA CAG CAC CCG GTA TTC	21-mer; antisense
5	U6 RNA	TGA CAC GCA AAT TCG TGA AGC	21-mer; antisense
6	miR-200c-3p	TCC ATC ATT ACC CGG CAG TAT TA	23-mer; antisense
7	miR-18a-5p	CTA TCT GCA CTA GAT GCA CCT TA	23-mer; antisense
8	miR-10b-5p	CAC AAA TTC GGT TCT ACA GGG TA	23-mer; antisense
9	miR-10b-3p	TAT TCC CCT AGA ATC GAA TCT G	22-mer; antisense
10	miR-381-3p	ACA GAG AGC TTG CCC TTG TATA	22-mer; antisense

## Data Availability

The data presented in this study are available on request from the corresponding author.

## Supplementary Materials

The following are available online at https://www.mdpi.com/article/10.3390/cimb43020036/s1. This article contains supporting information: (1) Supplementary Table S1. Comparison of current work with seminal published studies using conventional northern and liquid hybridization assays. (2) Supplementary File 1. Recipe of buffers and solutions used, and (3) Supplementary File 2. Full gel images.
