# Supplementary material for: A Comprehensive Analysis of Northern versus Liquid Hybridization Assays for mRNAs, Small RNAs, and miRNAs Using a Non-Radiolabeled Approach
Source: Curr Issues Mol Biol. 2021 Jun 22;43(2):457–84. doi: 10.3390/cimb43020036 (PMC8929067; doi:10.3390/cimb43020036)
Supplement: Supplementary file 1 [file cimb-43-00036-s001.zip › cimb-1198359-supplementary.pdf]

Supplementary Table S1

Comparison of current work with seminal published studies using conventional northern and liquid hybridization assays.

A Comprehensive Analysis of Northern versus Liquid Hybridization Assays for mRNAs, small RNAs, and miRNAs using a Non-radiolabeled Approach

Waqar Ahmad, Bushra Gull, Jasmin Baby, and Farah Mustafa

Department of Biochemistry, College of Medicine and Health Sciences, United Arab Emirates (UAE) University, Al Ain, UAE

Supplementary Table S1. Comparison of current work with seminal published studies using conventional northern and liquid hybridization assays.

**Research Article**	**Ahmad et al.,** (this study)	**Li F et al., 2016 (36)** ***Int J Mol Sci*** 17(9).	**Huang et al., 2014 (51)** ***Rice*** 7(1), 26.	**Li X et al., 2014. (47)** ***RNA*** 20(2), 252-9.	**Li X et al. 2012 (41)** ***Methods.*** 58(2), 151-5.	**Wang et al. 2010 (32) Int J Mol Sci.** 11(9), 3138-48.	**Kim et al. 2010 (35) Nucleic Acids Res** 38(7), e98.
**Paper Title**	A comprehensive analysis of northern *versus* liquid hybridization assays for mRNAs, small RNAs, and miRNAs using a non-radiolabeled approach	Liquid hybridization and solid phase detection: a highly sensitive and accurate strategy for microRNA detection in plants and animals	A non-radioactive method for small RNA detection by northern blotting	A convenient system for highly specific and sensitive detection of miRNA ex-pression	Detecting miRNAs by liquid hybridization and color development	Rapid and Accurate detection of plant miRNAs by liquid northern hybridization	A sensitive non-radioactive northern blot method to detect small RNAs
**Organism**	**Animal** (mouse HC11 cells)	**Plant** (rice) **Animal** (mouse tissue & human blood)	**Plant** (rice)	**Animal** (rat cells/tissues)	**Animal** (rat tissues)	**Plant** (rice, maize, wheat, tobacco, etc.)	**Animal** (human MCF-7 cells)
**Conventional /Liquid Hybridization**	Liquid and Conventional	Liquid and Conventional	Conventional	Liquid and Conventional	Liquid	Liquid	Conventional
**Target RNA**	mRNAs<br>small RNAs (5S & U6)<br>miRNAs	miRNAs	miRNAs<br>siRNAs	miRNAs	miRNAs	miRNAs	miRNAs
**RNA amount tested**	**Total RNA**<br>**NB:** 25-2.5 ug<br>**LH:** 1.0-0.25 ug	**Small RNA**<br>**NB:** NA<br>**LH:** 1 ug	**Total RNA**<br>1-20 ug<br>**Small RNA**<br>0.5-10 ug	**Total RNA**<br>**NB:** 20 ug<br>**LH:** 1ug<br>**Small RNA**<br>**LH:** 4 ng	**Small RNA**<br>8 ng	**Small RNA**<br>1 ug	**Total RNA**<br>3-10 ug

**Level of sensitivity**	**RNA**<br>**NB**: < 3.12 ug for mRNA; <3.12 ug for small RNAs; undetectable for miRNAs<br>**LH**: <0.312 ug for mRNA; <0.312 ug for small RNAs; <250 ng for miRNAs<br>**Probes:**<br>*mRNA:* 3 pmol (50 pmol/ml)<br>*small RNA & miRNAs*: 0.3 pmol (10 pmol/sample)	**Small RNA**<br>**NB:** NA<br>**LH:** 1ug<br>**Probe –**<br>*DIG-CDP Star:* 0.01–0.25 fmol<br>*Biotin-CDP Star*: 0.005–0.1 fmol<br>*Biotin-luminol:* 0.05–0.5 fmol	**Total RNA:** 1ug<br>**Small RNA:** 1ug<br><br>**Probe:** 50 pmol/ml	**RNA**<br>**NB**: 20 ug total RNA<br>**LH:**1 ug total RNA<br>**LH:** 4 ng small RNA	**Small RNA**<br>8 ng<br><br>**Probe:** 25 fmol	**Small RNA**<br>1 ug<br><br>**Probe:** 0.1 pmol	**Total RNA**<br>3 ug<br><br>**Probe:** 0.05 fmol
**Technique (Dot/PAGE)**	PAGE	PAGE	PAGE	Dot blot	Dot blot	Dot blot & PAGE	PAGE
**Probe type**	**For mRNA:** 5′ biotinylated dsDNA probe<br>**For small & miRNAs:** 5′ biotinylated oligo probe	5′ DIG probe or biotinylated probe	5′ or 3′ Biotin -labelled probe completely complementary	Hairpin DNA probes and 5′-biotinylated or γ-^{32}P-labeled oligonucleotides	5′ biotinylated probes	FITC-labelled probe complem-entary probe	DIG-labelled LNA or ^{32}P-labeled LNA probes
**Probe amount**	**NB:**<br>3 pmol (50 pmol/ml)<br>**LH:**<br>0.3 pmol (10 pmol/sample)	0.1 pmol	50 pmol/ml	**NB-** 5 pmol<br>**LH-** 20 fmol to 4 pmol	20 pmol	0.1 pmol (PAGE)<br>0.01 pmol (Dot)	0.05-0.5 nM (0.2 nM)<br>Difficult to detect 2 nM
**Enzyme used for detection**	Horseradish peroxidase	Alkaline Phosphatase or Horseradish peroxidase	Horseradish peroxidase	Alkaline Phosphatase	Alkaline Phosphatase or Horseradish Peroxidase	Florescence detection<br>No Enzyme Needed	Alkaline Phosphatase

**Crosslinking method**	**LH -** UV crosslinking (254 nm; 700 μJoules) for 5-7 mins & baked at 80°C for 2 hrs<br>**NB** - UV crosslinking (254 nm; 700 μJoules) for 5-7 mins & EDC-treated at 60°C for 2 hrs	UV Crosslinking (254 nm; 15–20 KJ/cm^2) for 5–9 mins	UV crosslinking (254 nm; 1,200 μjoules) for 20 mins & dried at 50°C for 30 mins	UV crosslinking (254 nm; 3,000 μjoules) for 90 secs	UV crosslinking (254 nm; 3,000 μjoules) for 90 secs	No Blotting	EDC crosslinking at 60’C for 1-2 hrs
**Detection type**	Chemiluminescence	Chemiluminescence	Chemiluminescence	**LH:** Colorimetric<br>**NB:** Phosphor imaging	Colorimetric	Fluorescence imaging	Phosphor imaging
**Detection method/ Instrument**	X- ray film detection<br><br>Sapphire and Typhoon Biomolecular Imagers	ChemiDoc XRS System	Chemiluminescent nucleic acid detection kit<br><br>X-ray film detection	Color development<br><br>X-ray film detection	Color development	CitaBlue conversion screen in a Chemi Doc XRS System	Phosphor image screen used in the ChemiDoc-IT Imaging System
**Substrate/ Reagent**	**Luminol based:**<br>**ECL:** X-Ray film detection & Typhoon biomolecular imager<br>**Femto:** Sapphire biomolecular imager	Luminol<br>CDP Star	Luminol	BCIP/NBP	BCIP/NBP<br>or<br>DAB<br>or<br>ECL Prime	Florescence detection.<br>No substrate needed.	CSPD
**Incubation time for detection**	**Luminol based:**<br>**ECL** – 2 min<br>**Femto** - 10 secs	30 mins	5 mins	X-ray film detection: 12 hrs BCIP/NBP: 2 mins	BCIP/NBP: 30 secs DAB: 5 secs ECL Prime: 1 min	NA	20 mins

**Exposure time**	5-30 secs	20 mins	Auto-exposure	5 secs – 1 min	5 sec – 15 mins	Auto-exposure	Autoexposure<br><br>DIG = 1 min<br>^{32}P = 24 h
**Conclusions**	Development of cost-effective, sensitive, and rapid biotin-based northern and liquid hybridization assays for mRNAs, small RNAs, and miRNAs	The development of Liquid Hybridization Solid Phase Detection (LHSPD) technique for analysis of miRNAs in plants and animals	Development of the Northern blot method by using biotin-labeled probes	An improved version of liquid hybridization and color development technique (LHCD) with signal amplification using bridged-PCR (named LH-CD or aLHCD)	Development of liquid hybridization and color development (LHCD) technique that uses signal amplification of avidin-biotin complex (ABC) for detection of miRNAs	Establishment of the Liquid Northern hybridization technique using fluorescently labeled oligonucleotide probes for small RNA detection	Development of a new northern blot-based protocol (LED) using digoxigenin (DIG)-labeled, LNA-probes and EDC cross-linking technique for small RNA detection

Supplementary File 1

Recipe of Buffers and Solutions Used

A Comprehensive Analysis of Northern versus Liquid Hybridization Assays for mRNAs, small RNAs, and miRNAs using a Non-radiolabeled Approach

Waqar Ahmad, Bushra Gull, Jasmin Baby, and Farah Mustafa

Department of Biochemistry, College of Medicine and Health Sciences,
United Arab Emirates (UAE) University, Al Ain, UAE

1% Formaldehyde-agarose (FA) gel
Agarose = 1.5 g
10x MOPS = 15 ml
RNase free H_2O = 135 ml
37% formaldehyde= 2.7 ml
EtBr = 5 µl (of 10 mg/ml)

15% Urea-acrylamide gel (15 ml)
40% acrylamide = 5.63 ml
Urea = 7.2 g
10X TBE = 1.5 ml
Warm solution at 55°C until urea dissolve.
Bring to room temperature and add:
H_2O = up to 15 ml
20% APS = 200 µl
TEMED = 10 µl

Streptavidin blocker solution (500 ml)
1X Casein stock (50 ml)
1% Casein/ TBS = 5 ml
20% SDS = 1.25 ml
1X TBS = 43.75 ml

Blocker preparation
1X Casein-TBS = 50 ml
20% SDS = 12.5 ml
10% TBS = 43.75 ml
H_2O = 394 ml

Blocker washing (500 ml)
1x TBS = 487.5 ml
20% SDS = 12.5 ml

Native-PAGE non-denaturing gel (25 ml)
40% acrylamide = 7.5 ml
10X TBE = 2.5 ml
H_2O = 15 ml
20% APS = 200 µl
TEMED = 10 µl

Native RNA/DNA dye (10 ml)
Glycerol = 5 ml
1M EDTA = 0.3 ml
H_2O = 4.7 ml
Xylene cyanole = 5-10 mg
Bromophenol blue = 5-10 mg

Native (Non-denaturing) MOPS gel (100 ml)
Agarose = 1 g
10X MOPS = 10 ml
H_2O = up to 100 ml
EtBr = 5 µl (10 mg/ml)

Denaturing RNA loading buffer
Formamide = 9.5 ml
0.25M EDTA = 500 µl
Xylene cyanole = 5-10 mg
Bromophenol blue = 5-10 mg

20xSSC (500 ml)
Sodium Chloride = 87.65 g
Sodium Citrate = 44.1 g
H_2O = up to 500 ml

Modified Church buffer (500 ml)
Sodium hydrogen phosphate = 25.56 g
Sodium dihydrogen phosphate = 8.4 g
0.5 M EDTA = 1 ml
20% SDS = 175 ml
H_2O = up to 500 ml

Low Stringency buffer (100 ml)
20X SSC = 10 ml
20X SDS = 0.5 ml
H_2O = 89.5 ml

High Stringency buffer (100 ml)
20X SSC = 0.5 ml
20X SDS = 0.5 ml
H_2O = up to 99 ml

0.2 M EDC crosslinking buffer (12 ml)
Methylimidazole = 122.5 µl
H_2O = 10 ml
Adjust pH to 7.5-8.5 using HCl (~25 µl)
EDC = 0.375 g
H_2O = up to 12 ml

Liquid hybridization buffer (10 ml)
10 mM EDTA = 200 µl
1M NaCl = 3 ml
1M Sodium Phosphate (pH = 8.0) = 300 µl
H_2O = up to 10 ml

1M Sodium Phosphate buffer pH 8 (stock)

Add 1.42 g $NaHPO_4$ in 10 ml H_2O (Solution A)
Add 1.38 g NaH_2PO_4 in 10 ml H_2O (Solution B)
Mix 4.66 ml of Solution A + 340 µl of Solution B

10X TBE

Tris = 108 g
Boric acid = 55 g
0.5M EDTA = 40 ml (pH = 8)

TE buffer; 10 mM Tris/1 mM EDTA (100 ml)

1 M Tris-HCl = 1 ml (pH = 7.4)
0.5 M EDTA = 0.2 ml
H_2O = up to 100 ml

Membrane stripping buffer

SDS = 0.5g
Water= 100ml
Boil and immerse membrane in it.

Supplementary File 2
Full gel images for Figures 4, 6, 12, & 13

A Comprehensive Analysis of Northern *versus* Liquid Hybridization Assays for mRNAs, small RNAs, and miRNAs using a Non-radiolabeled Approach

Waqar Ahmad, Bushra Gull, Jasmin Baby, and Farah Mustafa

Department of Biochemistry, College of Medicine and Health Sciences,

United Arab Emirates (UAE) University, Al Ain, UAE

Figure 4

Conventional Northern

RNA1 — 25ug, 12.5ug, 6.25ug, 3.12ug; L; RNA2 — 25ug, 12.5ug, 6.25ug, 3.12ug

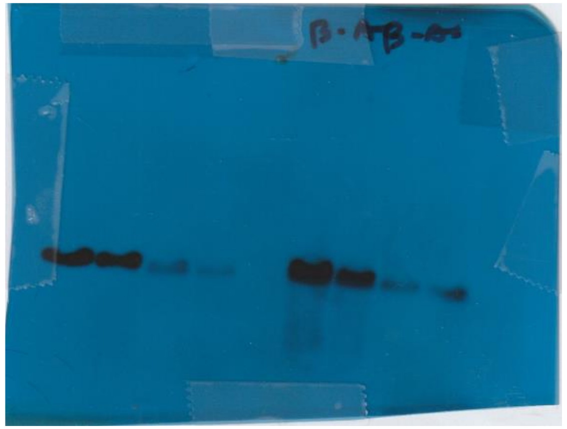

Beta-actin full image

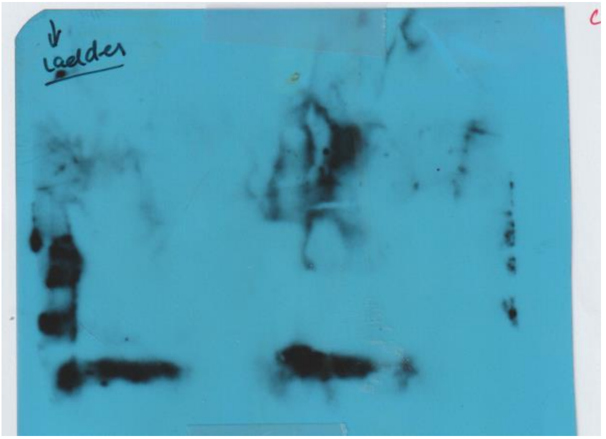

c-Myc full image

Liquid Hyb Northern

RNA1 — 25ug, 12.5ug, 6.25ug, 3.12ug; L; RNA2 — 3.12ug, 6.25ug, 12.5ug, 25 ug

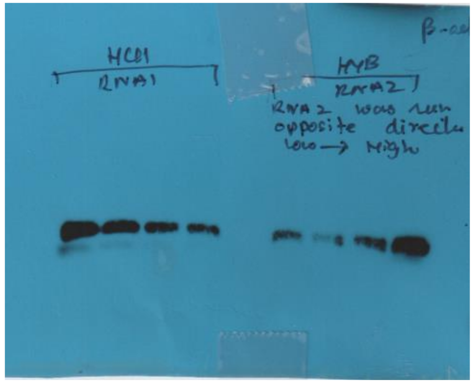

Beta-actin full image

RNA1 — 25ug, 12.5ug, 6.25ug, 3.12ug; L; RNA2 — 3.12ug, 6.25ug, 12.5ug, 25 ug

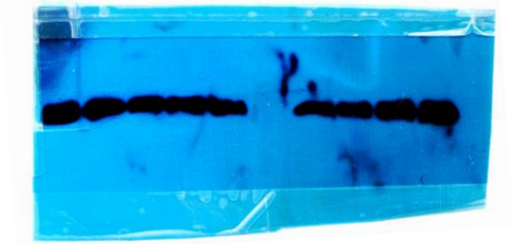

c-Myc full image

RNA2 in these images was run from lower concentrations to high. It was flipped in Figure 4(d) for the manuscript.

Figure 6

(a)

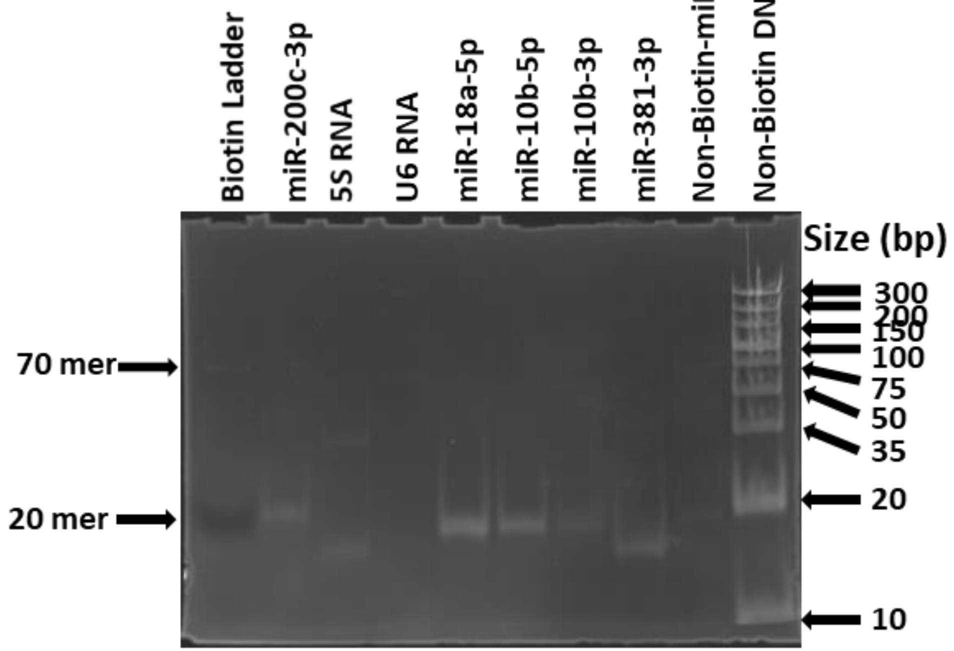

Ethidium Bromide

(c)

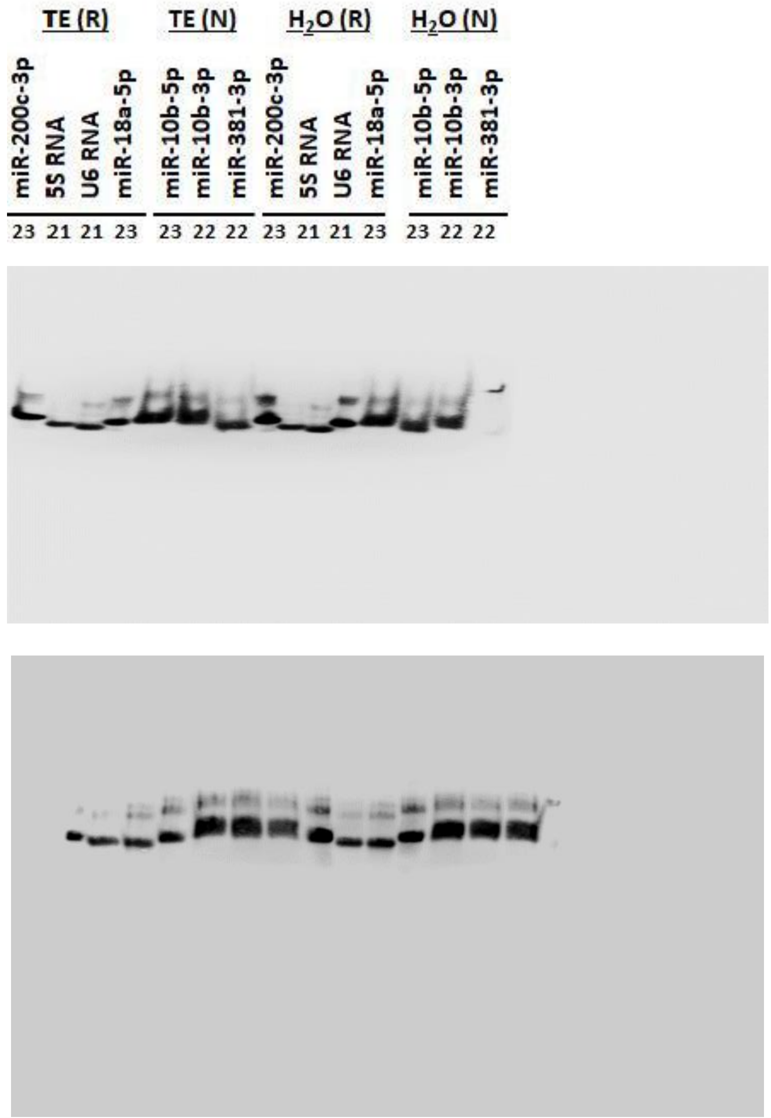

Sapphire

Figure 12

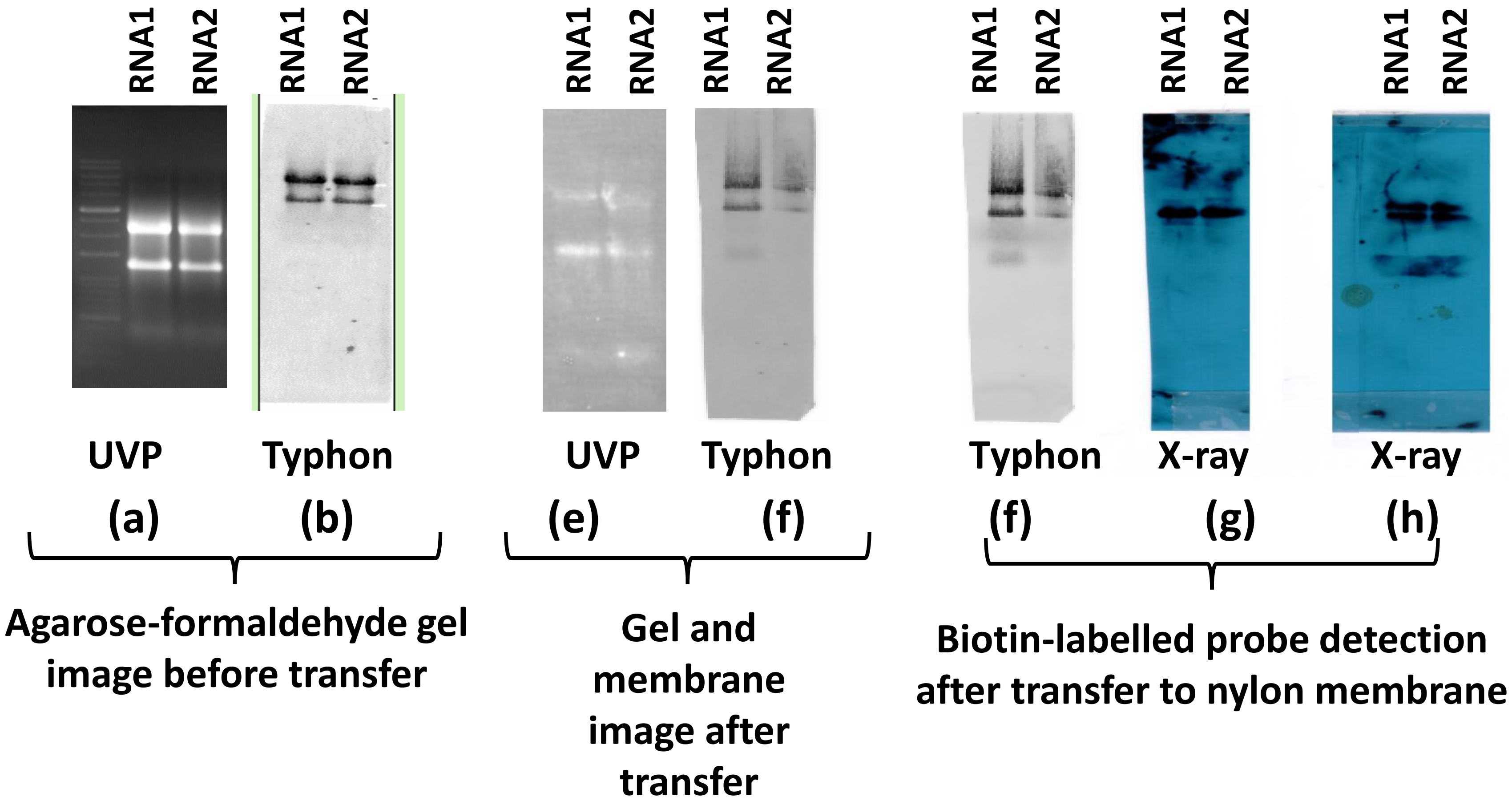

RNA1
RNA2
RNA1
RNA2
RNA1
RNA2
RNA1
RNA2
RNA1
RNA2
RNA1
RNA2
RNA1
RNA2
UVP
Typhon
UVP
Typhon
Typhon
X-ray
X-ray
(a)
(b)
(e)
(f)
(f)
(g)
(h)
Agarose-formaldehyde gel image before transfer
Gel and membrane image after transfer
Biotin-labelled probe detection after transfer to nylon membrane

Figure 13

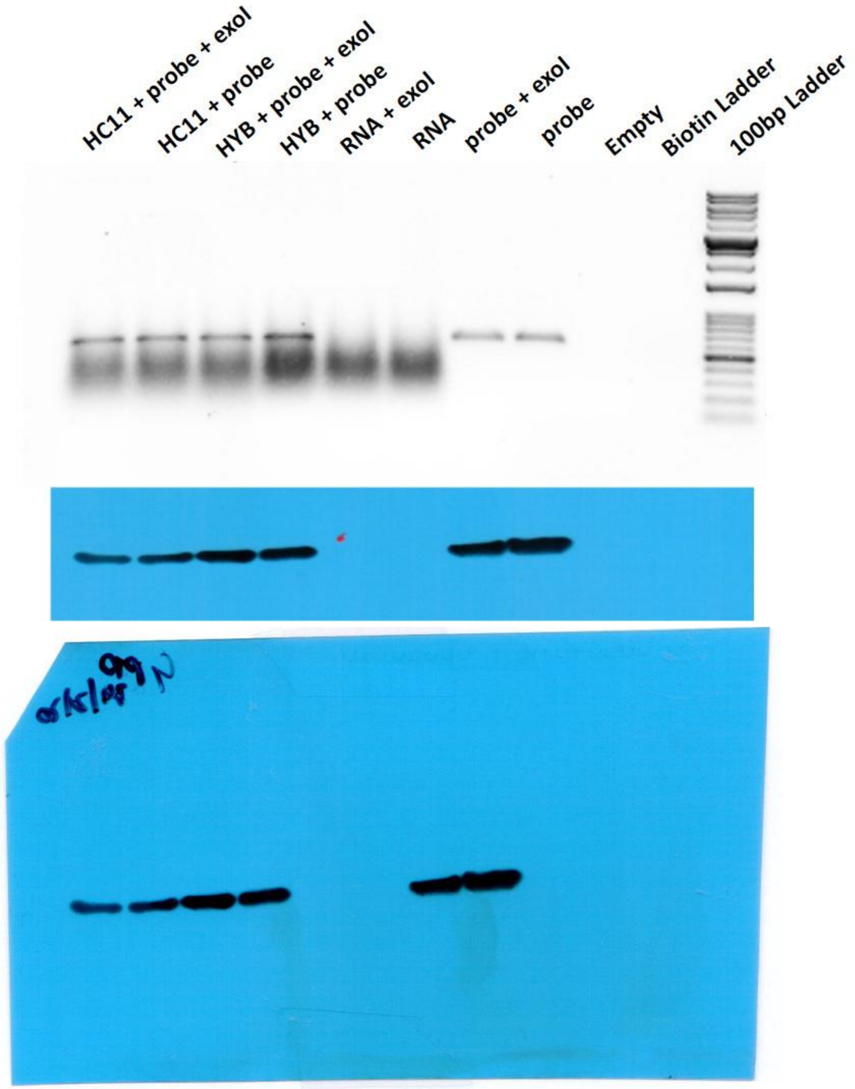

Full gel image

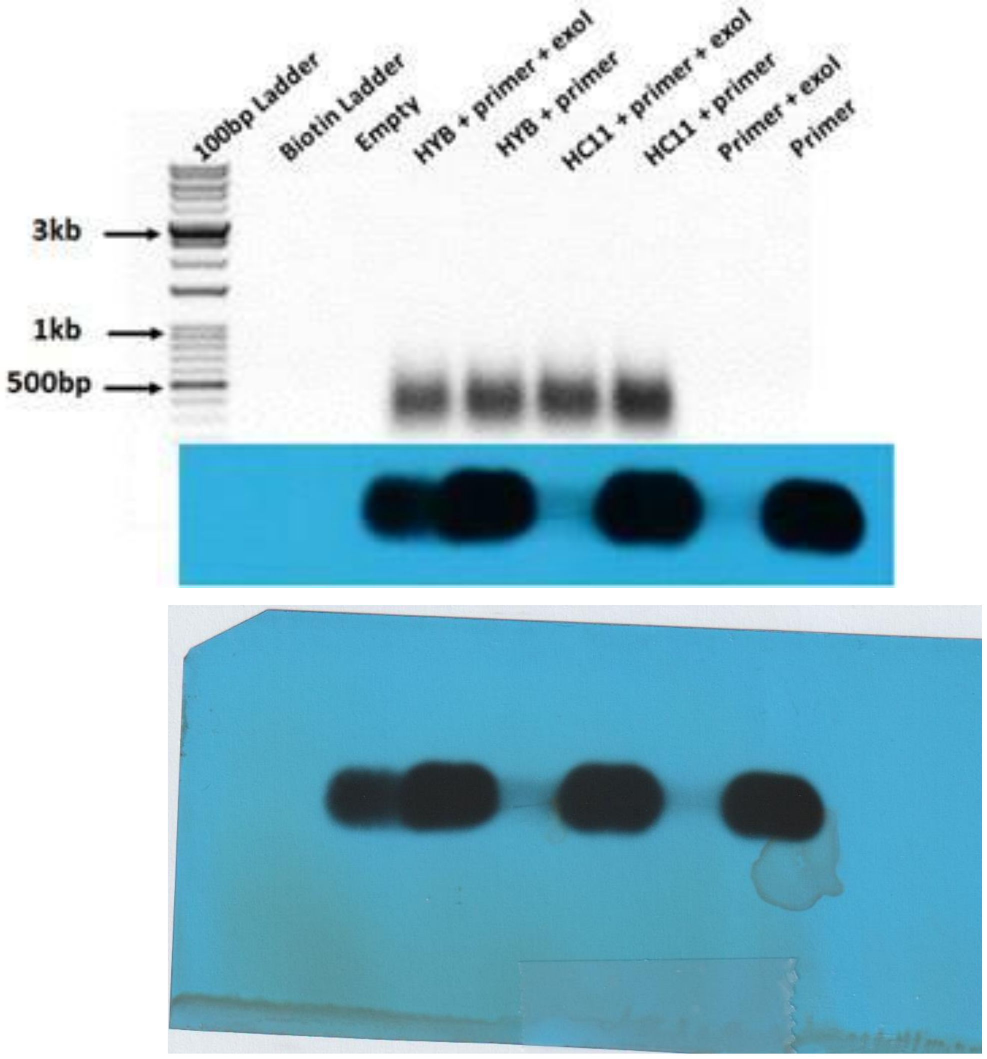

Full gel image
